# Novel daidzein analogs enhance osteogenic activity of bone marrow-derived mesenchymal stem cells and adipose-derived stromal/stem cells through estrogen receptor dependent and independent mechanisms

**DOI:** 10.1186/scrt493

**Published:** 2014-08-28

**Authors:** Amy L Strong, Jason F Ohlstein, Quan Jiang, Qiang Zhang, Shilong Zheng, Stephen M Boue, Steven Elliott, Jeffrey M Gimble, Matthew E Burow, Guangdi Wang, Bruce A Bunnell

**Affiliations:** Center for Stem Cell Research and Regenerative Medicine, Tulane University School of Medicine, 1430 Tulane Avenue, SL-99, New Orleans, LA 70112 USA; Department of Chemistry and RCMI Cancer Research Program, Xavier University of Louisiana, New Orleans, LA 70125 USA; US Department of Agriculture, Southern Regional Research Center, 1100 Robert E. Lee Blvd, New Orleans, LA 70124 USA; Department of Medicine, Tulane University School of Medicine, New Orleans, LA 70112 USA

## Abstract

**Introduction:**

Osteoporosis is a disease characterized by low bone mineral density (BMD) and increased risk of fractures. Studies have demonstrated the use of phytoestrogens, or plant-derived estrogens, such as genistein and daidzein, to effectively increase osteogenic activity of bone marrow-derived mesenchymal stem cells (BMSCs). Herein, the effects of daidzein analogs on the osteogenic differentiation efficiency of human BMSC and adipose-derived stromal/stem cells (ASC) were explored.

**Methods:**

BMSCs and ASCs underwent osteogenic differentiation in the presence of vehicle, 17β-estradiol (E2), phytoestrogens, or daidzein analogs. Cells were stained for alkaline phosphatase (ALP) enzymatic activity, calcium deposition by alizarin red s, and phosphate mineralization by silver nitrate. Gene expression analysis was conducted on cells treated with daidzein analogs.

**Results:**

Cells treated with E2, daidzein, or genistein increased calcium deposition by 1.6-, 1.5-, and 1.4-fold, respectively, relative to vehicle-treated BMSCs and 1.6-, 1.7-, and 1.4-fold relative to vehicle-treated ASCs, respectively. BMSCs treated with daidzein analog 2c, 2g, and 2l demonstrated a 1.6-, 1.6-, and 1.9-fold increase in calcium deposition relative to vehicle-treated BMSCs, respectively, while ASCs treated with daidzein analog 2c, 2g, or 2l demonstrated a 1.7-, 2.0-, and 2.2-fold increase in calcium deposition relative to vehicle-treated ASCs, respectively. Additional analysis with BMSCs and ASCs was conducted in the more efficient compounds: 2g and 2l. ALP activity and phosphate mineralization was increased in 2g- and 2l-treated cells. The analysis of lineage specific gene expression demonstrated increased expression of key osteogenic genes (RUNX2, c-FOS, SPARC, DLX5, SPP1, COL1A1, IGF1, SOST, and DMP1) and earlier induction of these lineage specific genes, following treatment with 2g or 2l, relative to vehicle-treated cells. Estrogen receptor (ER) inhibitor studies demonstrated that ER antagonist fulvestrant inhibited the osteogenic differentiation of 2g in BMSCs and ASCs, while fulvestrant only attenuated the effects of 2l, suggesting that 2l acts by both ER dependent and independent pathways.

**Conclusions:**

These studies provide support for exploring the therapeutic efficacy of daidzein derivatives for the treatment of osteoporosis. Furthermore, the patterns of gene induction differed following treatment with each daidzein analog, suggesting that these daidzein analogs activate distinct ER and non-ER pathways to induce differentiation in BMSCs and ASCs.

**Electronic supplementary material:**

The online version of this article (doi:10.1186/scrt493) contains supplementary material, which is available to authorized users.

## Introduction

Osteoporosis is a pathological condition associated with bone degeneration and is characterized by low bone mineral density (BMD) and alterations to the architecture of the bone. The low bone density and compromised architecture results in reduced bone strength and increased susceptibility to fractures, leading to significant morbidity and mortality [1–3]. While many factors contribute to the development of osteoporosis, age will probably be the leading risk factor due to the aging population in the United States [4]. It is estimated that more than 2 million people suffer from osteoporosis at a cost of $17 billion annually in the United States [5]. Although increasing physical activity is a modifiable lifestyle choice that can reduce the incidence of osteoporosis [6], the development of novel therapeutic interventions will further reduce the development of osteoporosis by supporting healthier bones over an individual’s lifetime.

Current treatment regimens for osteoporosis target bone regeneration or bone resorption, as these two processes are normally balanced in order to maintain strong, healthy bones. As such, therapeutic compounds have been divided into two groups: anti-resorptive drugs and anabolic drugs. Anti-resorptive drugs reduce the breakdown of bone during normal remodeling and reduce bone loss by limiting osteoclast activity [7]. These drugs include bisphosphonate, calcitonin, and denosumab. Studies have shown that delivery of these drugs independently or in combination is effective in reducing bone loss. While these drugs limit the severity of osteoporosis, it is still necessary for bone to undergo regeneration to restore the architecture of the bone and provide strength to the bones. Anabolic drugs have been shown not only to achieve higher BMD, but also to improve the quality and the strength of the bone [8].

Estrogens have anti-resorptive activity and anabolic activity, which have made them useful for the treatment of osteoporosis in postmenopausal women [9, 10]. However, the precise mechanism by which this occurs remains to be determined. Furthermore, while estrogens are considered powerful modulators of bone metabolism by reducing the development of osteoporosis and increasing BMD, their use in the form of hormone replacement therapy has been halted due to its association with an increased risk of developing breast and endometrial cancer [11–13]. Effective alternatives to estrogens are therefore necessary. Raloxifene, a selective estrogen receptor (ER) modulator, has been shown to produce estrogen-agonistic effects on bone and estrogen-antagonistic effects on uterine, endometrium, and breast tissue [14]. However, raloxifene has also been associated with increased risk of thromboembolic events [15]. There thus remains a need to identify superior pharmacological therapies to treat osteoporosis.

Plant-derived estrogens, or phytoestrogens, have gained significant attention and interest because these compounds have been shown to increase osteogenesis without increasing the risk of developing cancer [16–18]. More specifically, phytoestrogens isolated from soy, namely genistein and daidzein, are able to inhibit the bone resorption activity of osteoclasts while simultaneously stimulating osteogenic differentiation and maturation of bone marrow-derived mesenchymal stem cells (BMSCs) and osteoblasts [19]. In addition, previous studies have shown that these compounds have the ability to induce osteogenesis in osteoporosis-induced ovariectomized animal models [20].

While these studies are encouraging, the osteogenic potential of these phytoestrogens is less potent than the estrogens. Developing compounds with increased osteogenic potential without the tumorigenic potential of estrogens therefore remains a priority. Previously, our group designed and synthesized daidzein analogs that exhibited a wide range of estrogenic, anti-estrogenic, and osteogenic activity [21, 22]. Herein, the impact of these daidzein analogs on the osteogenic potential of human BMSCs and adipose derived stromal/stem cells (ASCs) is described. Although previous studies have focused on BMSCs as the precursor to osteoblasts, recent studies have shown that ASCs differentiate into osteoblasts [23, 24]. Additionally, the ease in isolation of ASCs and their abundance from adipose tissue makes them ideal candidates for tissue engineering and regenerative purposes.

To characterize the effect of these daidzein analogs (2c, 2g, and 2l) on BMSCs and ASCs, treated cells were assessed for the amount of calcium deposition by alizarin red staining, alkaline phosphatase (ALP) activity by 5-bromo-4-chloro-3-indolyl phosphate (BCIP/NBT) staining, and phosphate mineralization by silver nitrate staining. Furthermore, the dose of these compounds necessary to elicit 50% of the maximal osteogenic effect (EC_50_) on BMSCs and ASCs was identified and their efficacy and potency was compared with 17β-estradiol (E2). To determine the downstream targets of these compounds, mRNA expression analysis was conducted on key osteogenic factors. Together, these studies provide support for daidzein analogs as novel therapeutic compounds that have the potential to reduce osteoporosis through increasing osteogenesis.

## Materials and Methods

### Materials

Anti-CD45-PeCy7, anti-CD11b-PeCy5, anti-CD166-phycoerythrin, anti-CD105-phycoerythrin, anti-CD90-PeCy5, anti-CD34-phycoerythrin, isotype control fluorescein isothiocyanate human IgG_1_, and isotype-control phycoerythrin human IgG_2a_ were purchased from Beckman Coulter (Indianapolis, IN, USA). Anti-CD44-allophycocyanin was purchased from BD Biosciences (San Jose, CA, USA). Type 1 collagenase, bovine serum albumin (fraction V), calcium chloride, dexamethasone, isobuytlmethylxanthine, indomethacin, ascorbate 2-phosphate, β-glycerol phosphate, alizarin red S, oil red O, cetylpyridinium chloride, BCIP/NBT, silver nitrate, E2, daidzein, and genistein were purchased from Sigma (St Louis, MO, USA). Daidzein analogs (i.e. 2c, 2g, and 2l) were synthesized in our laboratory as described previously [21, 22].

### Human subjects

Primary human BMSCs were obtained from six healthy consenting Caucasian female donors under a protocol approved by Tulane University Institutional Review Board. The cells were prepared from bone marrow aspirates taken from the iliac crest of six individuals. Nucleated cells were isolated using Ficoll-Paque density gradient (Amersham Pharmacia Biotech, Milwaukee, WI, USA) and resuspended in complete culture media (CCM), which consisted of α-Minimal Essential Medium (Gibco, Grand Island, NY, USA), 20% fetal bovine serum (Atlanta Biologicals, Lawrenceville, GA, USA), 100 units/ml penicillin/100 μg/ml streptomycin (Gibco), and 2 mM l-glutamine (Gibco). The cells were then seeded on a 150 cm^2^ culture dish (Nunc, Rochester, NY, USA) and maintained in a humidified 5% carbon dioxide (CO_2_) incubator at 37°C. Medium was changed every 3 to 4 day. When the cultures reached 70% confluence, the cells were harvested with 0.25% trypsin/1 mM ethylenediamine tetraacetic acid (EDTA; Gibco) and cryopreserved prior to experimental use.

Primary human ASCs were obtained from six healthy consenting Caucasian female donors undergoing elective liposuction procedures under a protocol approved by Pennington Biomedical Research Center Institutional Review Board. ASCs were isolated from processed lipoaspirates from the subcutaneous adipose tissue of subjects. Lipoaspirates were incubated in 0.1% type I collagenase and 1% bovine serum albumin dissolved in 100 ml phosphate-buffered saline (PBS) supplemented with 2 mM calcium chloride. The mixture was placed in a 37°C shaking water bath at 75 rpm for 60 minutes and then centrifuged to remove oil, fat, primary adipocytes, and collagenase solution, leaving behind a pellet of cells. Cells were resuspended in CCM, plated on 150 cm^2^ culture dishes, and maintained in a humidified 5% CO_2_ incubator. Fresh medium was added every 2 to 3 days until cells achieved 80 to 90% confluence and were harvested with 0.25% trypsin/1 mM EDTA and cryopreserved prior to experimental use.

### Cell culture

Frozen vials of approximately 10^6^ BMSCs or ASCs were thawed, plated onto 150 cm^2^ culture dishes (Nunc) in 20 ml CCM and incubated at 37°C with 5% humidified CO_2_. After 24 hours, medium was removed and adherent, viable cells were washed with PBS, harvested with 0.25% trypsin/1 mM EDTA (Gibco), and replated at 100 cells/cm^2^ in CCM. Medium was replaced every 3 to 4 days. For all experiments, subconfluent cells (<70% confluence) between passages 2 and 6 were used.

### Flow cytometry

BMSCs and ASCs were harvested with 0.25% trypsin/1 mM EDTA for 3 to 4 minutes at 37°C. A total of 3 × 10^5^ cells were suspended in 50 μl PBS and incubated with fluorescence-labeled primary antibodies. The samples were incubated for 30 minutes at room temperature and washed with PBS. The samples were then analyzed with Galios Flow Cytometer (Beckman Coulter, Brea, CA, USA) running Kaluza software (Beckman Coulter). To assay cells by forward and side scatter, FACScan was standardized with microbeads (Dynosphere uniform microspheres; Bangs Laboratories Inc., Thermo Scientific, Waltham, MA, USA). At least 10,000 events were analyzed and compared with isotype controls.

### Colony-forming unit assay

BMSCs and ASCs were plated at a density of 100 cells on a 10 cm^2^ plate in CCM and incubated for 14 days. Plates were then rinsed with PBS and stained with 3% crystal violet (Sigma) for 30 minutes at room temperature. Plates were washed with PBS and once with tap water. Colonies that were larger than 2 mm in diameter were counted.

### Differentiation protocols

#### Osteogenic differentiation

BMSCs and ASCs were cultured in six-well plates in CCM until 70% confluence. Medium was replaced with fresh osteogenic differentiation medium (ODM) consisting of 50 μM ascorbate 2-phosphate, 10 mM β-glycerol phosphate, and 10 nM dexamethasone. Where indicated, the fetal bovine serum in ODM was substituted for charcoal dextran-stripped fetal bovine serum (charcoal dextran-stripped osteogenic differentiation medium (CDS-ODM); Atlanta Biologicals).

#### Adipogenic differentiation

BMSCs and ASCs were cultured in six-well plates in CCM until 70% confluence. Medium was replaced with fresh adipogenic induction media made with CCM supplemented with 0.5 μM dexamethasone, 0.5 mM isobuytlmethylxanthine, and 50 μM indomethacin. Where indicated, the fetal bovine serum in adipogenic differentiation medium was substituted for charcoal dextran-stripped adipogenic differentiation medium (CDS-ADM).

### Phytoestrogen, daidzein analog, and fulvestrant treatment

BMSCs and ASCs were plated in CCM and allowed to adhere to plastic. The medium was replaced with CDS-ODM or CDS-ADM and supplemented with vehicle, 10 nM E2, 1 μM daidzein, 1 μM genistein, or 1 μM daidzein analog 2c, 2g, or 2l for 14 days. Furthermore, where indicated, cells were treated with CDS-ODM and supplemented with log-fold increases of E2, daidzein, or daidzein analog from 100 pM to 1 mM. ER antagonist studies were conducted by concurrent treatment with 100 nM fulvestrant (ICI187,280; Sigma) and vehicle, 10 nM E2, 1 μM daidzein, or 1 μM daidzein analog 2g or 2l in CDS-ODM.

### Staining and quantification protocols

#### Alizarin red staining and quantification

After 14 days, cells undergoing osteogenic differentiation in ODM or CDS-ODM were fixed in 10% formalin for 1 hour, washed with distilled water, and stained with 1% alizarin red (pH 4.1) to visualize calcium deposition in the extracellular matrix as a marker of early osteogenesis. Images were acquired at 4× magnification on a Nikon Eclipse TE200 with a Nikon Digital Camera DXM1200F using Nikon ACT-1 software (Melville, NY, USA). For quantification, alizarin red was extracted from each well with 10% cetylpyridinium chloride and read at 584 nm (FLUOstar optima; BMG Labtech, Durham, NC ). To normalize to the amount of protein in each sample, protein extraction with RIPA buffer (Pierce, Thermo Scientific) and protein quantification with the BCA assay (Thermo Scientific) were performed according to manufacturer’s instructions.

#### Oil Red O staining and quantification

After 14 days, cells undergoing adipogenic differentiation in adipogenic differentiation medium or CDS-ADM were fixed in 10% formalin for 1 hour, and stained with oil red O, composed of two parts PBS and three parts 0.5% oil red O stock solution to visualize neutral lipids. Images were acquired at 10× magnification on a Nikon Eclipse TE200 with a Nikon Digital Camera DXM1200F using the Nikon ACT-1 software. For quantification, oil red O was extracted from each well with isopropanol and read at 544 nm (FLUOstar optima). To normalize to the amount of protein in each sample, protein extraction with RIPA buffer (Pierce, Thermo Scientific) and protein quantification with the BCA assay (Thermo Scientific) were performed according to manufacturer’s instructions.

#### BCIP/NBT staining

After 3 days, cells undergoing osteogenic differentiation in CDS-ODM were fixed in 10% formalin for 1 hour, washed with distilled water, and incubated in BCIP/NBT to visualize ALP activity. Images were acquired at 4× magnification on a Nikon Eclipse TE200 with a Nikon Digital Camera DXM1200F using Nikon ACT-1 software.

#### Silver nitrate staining

After 14 days, cells undergoing osteogenic differentiation in CDS-ODM were fixed in 10% formalin for 1 hour, washed with distilled water, and incubated in 3% silver nitrate to visualize phosphate mineralization in the extracellular matrix as a late marker of osteogenesis. Images were acquired at 4× magnification on a Nikon Eclipse TE200 with a Nikon Digital Camera DXM1200F using Nikon ACT-1 software.

### MTT assay

Assessment of cell viability was performed with the MTT assay. BMSCs and ASCs were plated in triplicate in 96-well plates (500 cells/well) in CCM supplemented with vehicle, daidzein analog 2g, or daidzein analog 2l. After 7 and 14 days, cells were incubated with 10 mM MTT (Invitrogen, Grand Island, NY, USA) for 4 hours at 37°C with 5% humidified CO_2_. A total of 100 μl dissolving solution (10% SDS, 0.01 M HCl) was added to each well and incubated for 12 to 16 hours at 37°C. Absorbance was measured at 544 nm (FLUOstar optima).

### RNA isolation, cDNA synthesis, quantitative reverse transcription polymerase chain reaction

Cells treated with vehicle, 10 nM E2, 1 μM daidzein, or 1 μM daidzein analog in CDS-ODM were collected on days 3, 7, and 14. Where indicated, BMSCs and ASCs treated with vehicle, E2, daidzein, or daidzein analog were simultaneously treated with 100 nM fulvestrant and collected on days 3, 7, and 14. Total cellular RNA was extracted from BMSCs and ASCs using the RNeasy Mini Kit (Qiagen, Valencia, CA, USA), purified with DNase I digestion (Invitrogen) according to the manufacturer’s instructions, and reverse transcribed using the SuperScript VILO cDNA synthesis kit (Invitrogen). Quantitative real-time polymerase chain reaction was performed using the EXPRESS SYBR GreenER qPCR SuperMix Kit (Invitrogen) according to the manufacturer’s instructions. The following forward and reverse primer sequences were used to detect changes in gene expression: runt-related transcription factor 2 (RUNX2), 5′-CTCACTACCACACCTACCTG-3′ and 5′-TCAATATGGTCGCCAAACAGATTC-3′; FBJ murine osteosarcoma viral oncogene homolog (c-Fos), 5′-CCTGTCAAGAGCATCAGCAG-3′ and 5′-GTCAGAGGAAGGCTCATTGC-5′; osteonectin (SPARC), 5′-TGTGGGAGCTAATCCTGTCC-3′ and 5′-TCAGGACGTTCTTGAGCCAGT-3′; distal-less homeobox 5 (DLX5), 5′-TGGCCCGAGTCTTCAGCTAC′ and 5′-TGGTTGGTCGGTCTCTTTCT-3′; secreted phosphoprotein 1 (SPP1), 5′-GCTCTAGAATGAGAATTGCACTG-3′ and 5′-TGTCGGTCCTGAGGTAACTG-3′; collagen type 1 alpha (COL1A1), 5′-CATGTTCAGCTTTGTGGACCTC-3′ and 5′-AGGTGATTGGTGGGATGTCTT-3′; insulin-like growth factor 1 (IGF1), 5′-CTGTGATCTAAGGAGGCTG-3′ and 5′-TTCGTGTTCTTGTTGGTAGA-3′; dentin matrix acidic phosphoprotein 1 (DMP1), 5′-GTGAGTGAGTCCAGGGGAGATAA-3′ and 5′-TTTTGAGTGGGAGAGTGTGTGC-3′; sclerostin (SOST), 5′-TCCCCACCACCCCTTTG-3′ and 5′-GGTCACGTAGCGGGTGAA-3′; and β-actin, 5′-CACCTTCTACAATGAGCTGC-3′ and 3′-TCTTCTCGATGCTCGACGGA-3′. All reverse transcription-polymerase chain reaction primers were designed using Primer3 (Free Software Foundation Inc.,Boston, MA, USA) and purchased from Integrated DNA Technologies (Coralville, IA, USA). The expression of human β-actin was used to normalize the mRNA content. Samples were tested in triplicate. No-template controls and no-reverse transcription controls were included in each polymerase chain reaction run.

### Statistical analysis

All values are presented as the mean ± standard deviation. Experiments were conducted separately with each donor, plated in triplicates for technical duplicates. The values for the technical duplicates for each donor were averaged together. The values for the six donors were averaged together to calculate the mean ± standard deviation. The statistical differences between two or more groups were determined by analysis of variance, followed by *post hoc* Bonferroni multiple comparison tests. Statistical significant was set at *P* < 0.05. Only statistically significant values were considered relevant. Analysis was performed using Prism (Graphpad Software, San Diego, CA, USA).

## Results

### BMSCs and ASCs demonstrate similar stem cell characteristics

BMSCs and ASCs were stained for cell surface antigens, plated for colony-forming units, and induced to differentiate down osteogenic and adipogenic lineages. BMSCs and ASCs displayed overlapping cell surface marker profiles (CD44^+^, CD90^+^, CD105^+^, CD166^+^, CD11b^−^, CD34^−^, and CD45^−^), were able to form colony-forming units, and underwent osteogenic and adipogenic differentiation (Additional file 1).

### Phytoestrogens enhance osteogenic differentiation while only estradiol enhanced adipogenic differentiation of BMSCs and ASCs

**Figure 1:**
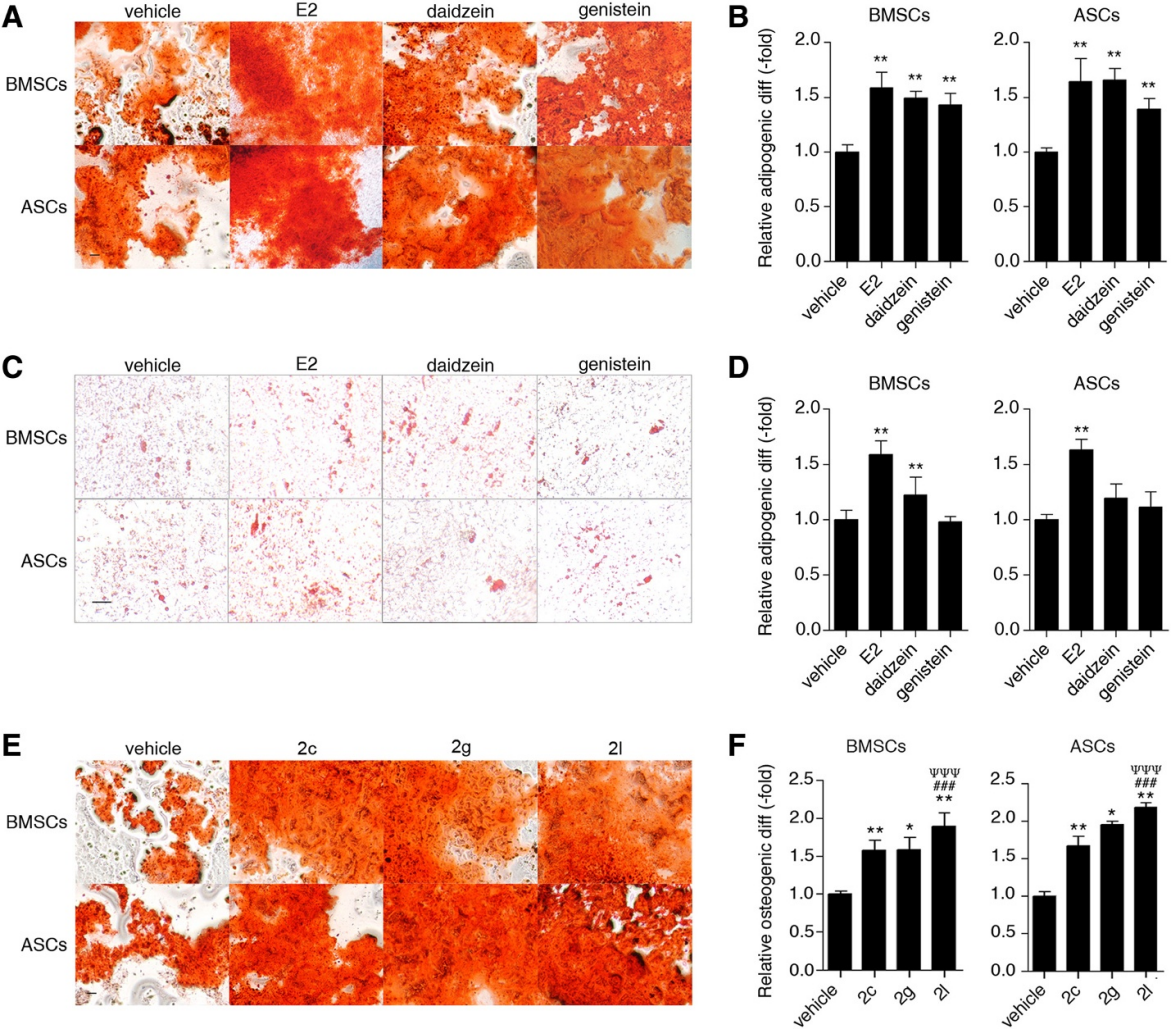
**Estradiol and phytoestrogens enhance osteogenic and adipogenic differentiation of bone marrow-derived mesenchymal stem cells and adipose-derived stromal/stem cells. (A)** to **(D)** Bone marrow-derived mesenchymal stem cells (BMSCs; *n* = 6) and adipose-derived stromal/stem cells (ASCs; *n* = 6) were cultured in charcoal dextran-stripped osteogenic differentiation medium (CDS-ODM) or charcoal dextran-stripped adipogenic differentiation medium (CDS-ADM) and simultaneously delivered vehicle (dimethylsulfoxide (DMSO)), 17β-estradiol (E2; 10 nM), daidzein (1 μM), or genistein (1 μM). **(A)** After 14 days, cells treated with E2, daidzein, or genistein and induced with CDS-ODM were stained with alizarin red. Scale bar represents 100 μm. **(B)** Stains were eluted with 10% cetylpyridinium chloride (CPC) to quantify the amount of alizarin red staining and measured at 590 nm. Osteogenic differentiation was determined relative to vehicle-treated cells (normalized to 1.0). **(C)** After 14 days, cells induced with CDS-ADM were stained with oil red O. Scale bar represents 100 μm. **(D)** To quantify the amount of oil red O staining, cells were eluted with isopropanol and measured at 544 nm. Adipogenic differentiation was determined relative to vehicle-treated cells (normalized to 1.0). **(E)**, **(F)** BMSCs (*n* = 6) and ASCs (*n* = 6) were cultured in CDS-ODM and simultaneously delivered vehicle (DMSO), E2 (10 nM), daidzein (1 μM), daidzein analog 2c (1 μM), daidzein analog 2g (1 μM), or daidzein analog 2l (1 μM). **(E)** After 14 days, cells induced with CDS-ODM were stained with alizarin red. Scale bar represents 100 μm. (F) To quantify the amount of alizarin red staining in BMSCs and ASCs treated with vehicle, E2, daidzein, or daidzein analogs, cells were eluted with 10% CPC and measured at 590 nm. Osteogenic differentiation was determined relative to vehicle-treated cells normalized to 1.0. Bars, ± standard deviation. *P < 0.05; **P < 0.01 compared with vehicle-treated cells. ^###^ *P* < 0.001 compared with E2-treated cells. ^ΨΨΨ^ *P* < 0.001 compared with daidzein-treated cells.

BMSCs and ASCs were cultured in CDS-ODM and supplemented with vehicle, E2, daidzein, or genistein. After 14 days, cells were stained with alizarin red and imaged with bright-field microscopy (Figure 1A). Alizarin red staining was quantified by eluting the alizarin red staining and acquiring optical density measurements. E2-treated, daidzein-treated, and genistein-treated BMSCs demonstrated 1.6-fold, 1.5-fold, and 1.4-fold greater osteogenic differentiation compared with vehicle-treated BMSCs, respectively (*P* < 0.01; Figure 1B). ASCs treated with E2, daidzein, and genistein demonstrated a 1.6-fold, 1.7-fold, and 1.4-fold increase in osteogenic differentiation relative to vehicle-treated ASCs (normalized to 1.0), respectively (*P* < 0.01; Figure 1B). It should be noted that while E2, daidzein, and genistein all enhanced osteogenic differentiation on average, two of the six BMSC donors and three of the six ASC donors did not respond to genistein treatment. Furthermore, visualization with bright-field microscopy did not demonstrate enhanced cell death in genistein-treated BMSCs or ASCs, compared with vehicle-treated cells.

To determine the effect of E2 or phytoestrogens on adipogenic differentiation of BMSCs and ASCs, cells were cultured in CDS-ADM and supplemented with E2, daidzein, and genistein. After 14 days, cells were stained with oil red O and imaged with bright-field microscopy (Figure 1C). Oil red staining was quantified by eluting with isopropanol and measuring the optical density of the extracted product. E2-treated BMSCs and ASCs demonstrated enhanced adipogenic differentiation by 1.6-fold compared with vehicle-treated BMSCs and ASCs (*P* < 0.01; Figure 1D). Daidzein enhanced adipogenic differentiation of BMSCs by 1.2-fold compared with vehicle-treated BMSCs (*P* < 0.01; Figure 1D) but had no effect on the adipogenic differentiation of ASCs. Genistein also had no effect on the adipogenic differentiation of BMSCs or ASCs.

### Daidzein analogs enhance osteogenic differentiation while reducing adipogenic differentiation of BMSCs and ASCs

To determine the osteogenic potential of daidzein analogs on BMSCs and ASCs, daidzein analogs 2c, 2g, and 2l were supplemented into CDS-ODM and at each media change. Cells were then stained with alizarin red and imaged with bright-field microscopy (Figure 1E), and the amount of staining was quantified by eluting with cetylpyridinium chloride. BMSCs treated with analogs 2c, 2g, and 2l demonstrated a 1.6-fold, 1.6-fold, and 1.9-fold increase in osteogenesis, respectively, relative to vehicle-treated BMSCs (*P* < 0.05; Figure 1F). ASCs treated with analogs 2c, 2g, and 2l demonstrated a similar trend and osteogenesis was enhanced by a 1.7-fold, 2.0-fold, and 2.2-fold increase, respectively, relative to vehicle-treated ASCs (*P* < 0.05; Figure 1F). Furthermore, analog 2l-treated BMSCs and ASCs demonstrated enhanced osteogenesis compared with E2-treated and daidzein-treated BMSCs and ASCs (*P* < 0.001; Figure 1F), suggesting that analog 2l is a more potent osteogenic compound than E2 or daidzein.

While analog 2c-treated, 2g-treated, and 2l-treated BMSCs and ASCs demonstrated enhanced osteogenic differentiation, these compounds failed to simulate adipogenic differentiation. BMSCs and ASCs treated with analog 2c demonstrated adipogenic differentiation comparable with vehicle-treated BMSCs and ASCs, while analog 2g-treated and 2l-treated BMSCs and ASCs demonstrated a significant reduction in adipogenic differentiation compared with vehicle-treated, E2-treated, or daidzein-treated BMSCs or ASCs (*P* < 0.001; Additional file 2).

To assess whether changes observed with differentiation were associated with changes caused by cytotoxicity or increased proliferation, BMSCs and ASCs were cultured in CCM supplemented with vehicle, daidzein analog 2g, or daidzein analog 2l and assessed after 7 and 14 days. Analog 2g-treated and 2l-treated BMSCs demonstrated similar growth rates to vehicle-treated BMSCs (*P* > 0.05; Additional file 3). Likewise, 2g-treated and 2l-treated ASCs demonstrated similar rates of proliferation compared with vehicle-treated ASCs, as no difference was observed in cell number after 7 or 14 days (*P* > 0.05; Additional file 3).

### Daidzein analogs 2g and 2l have similar EC_50_ values but differ in effectiveness

**Figure 2:**
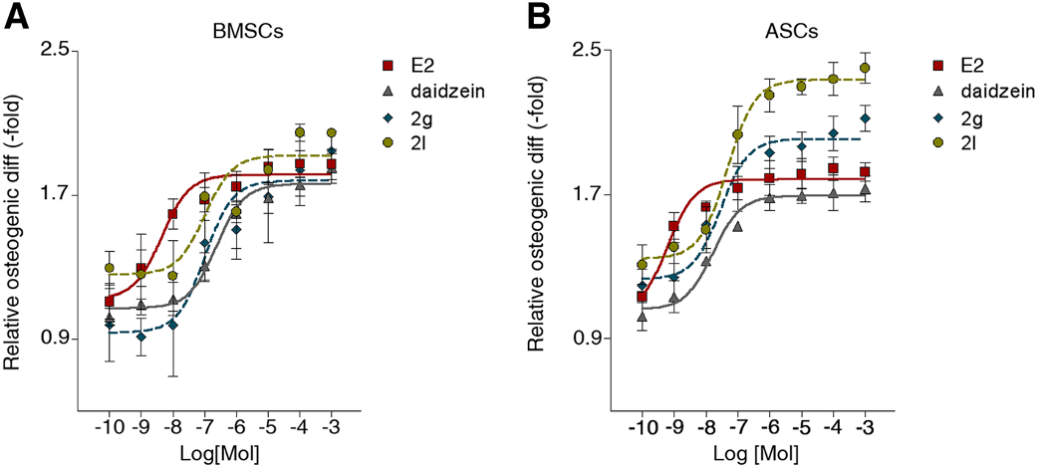
**Effect of daidzein analogs on osteogenic differentiation of bone marrow-derived mesenchymal stem cells and adipose-derived stromal/stem cells is dose dependent. (A)** Bone marrow-derived mesenchymal stem cells (BMSCs; *n* = 6) and **(B)** adipose-derived stromal/stem cells (ASCs; *n* = 6) were cultured in charcoal dextran-stripped osteogenic differentiation medium and simultaneously treated with vehicle, 17β-estradiol (E2), daidzein, or daidzein analog 2g or 2l at concentrations between 100 pm and 1 mM. After 14 days, cells were fixed, stained with alizarin red, destained with cetylpyridinium chloride, and measured at 590 nm. Osteogenic differentiation was determined relative to vehicle-treated cells normalized to 1.0. Bars, ± standard deviation.

Additional studies were conducted to determine the concentration of daidzein analogs required to induce EC_50_ on osteogenesis of BMSCs and ASCs in the most potent compounds: 2g and 2l. Analogs 2g and 2l were thus administered to BMSCs and ASCs at concentrations ranging from 100 pM to 1 mM, at log-fold increases. The EC_50_ value for E2 in BMSCs and ASCs was 10^-8.35^ and 10^-9.20^, respectively (Additional file 4). The EC_50_ value for daidzein, analog 2g, and analog 2l in BMSCs was 10^-6.62^, 10^-6.99^, and 10^-6.99^, respectively (Additional file 4). The EC_50_ values for daidzein, analog 2g, and analog 2l in ASCs were comparable with those for BMSCs: 10^-7.76^, 10^-7.53^, and 10^-7.32^, respectively (Additional file 4). E2 displays the most potent stimulation of osteogenesis in BMSCs and ASCs, leading to enhanced osteogenesis at 1 nM to 10 nM concentrations. However, analog 2l treatment at1 μM concentration resulted in the greatest degree of osteogenic differentiation in BMSCs (2.0-fold) and ASCs (2.4-fold; Figure 2). Higher doses of E2 were unable to induce osteogenesis equal to or exceeding that of analog 2l (*P* < 0.05; Figure 2).

### Enhanced alkaline phosphatase activity and increased phosphate deposition was observed in analog 2g-treated and 2l-treated BMSCs and ASCs

**Figure 3:**
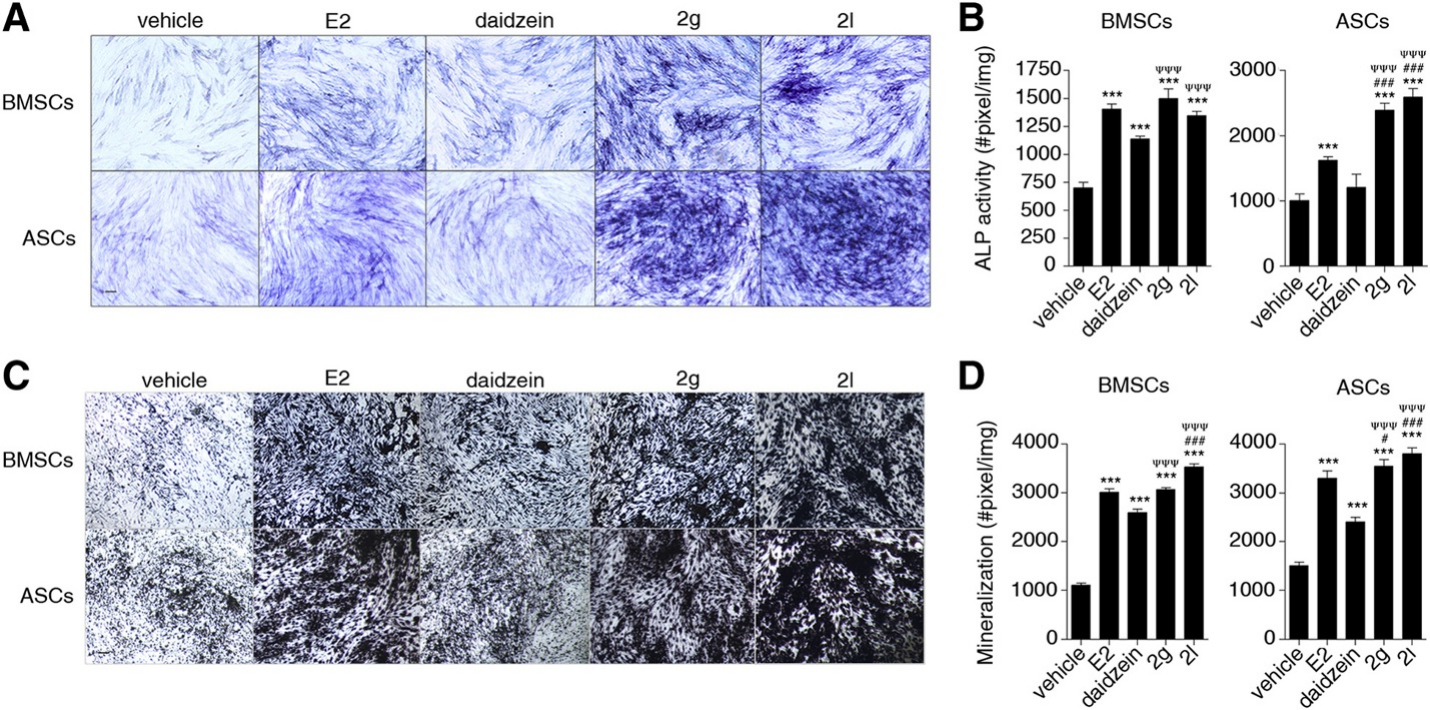
**Daidzein analogs increase alkaline phosphatase activity and phosphate deposition in bone marrow-derived mesenchymal stem cells and adipose-derived stromal/stem cells.** Bone marrow-derived mesenchymal stem cells (BMSCs; *n* = 6) and adipose-derived stromal/stem cells (ASCs; *n* = 6) were cultured in charcoal dextran-stripped osteogenic differentiation medium and simultaneously delivered vehicle, 17β-estradiol (E2; 10 nM), daidzein (1 μM), or daidzein analog 2g or 2l (1 μM). **(A)** After 3 days, cells were stained were fixed and stained with 5-bromo-4-chloro-3-indolyl phosphate. **(B)** To quantify alkaline phosphatase (ALP) activity, the number of positive pixels per image (for a total of five images/donor) was counted by Image J (Sigma, St. Louis, MO, USA). **(C)** After 14 days, cells were stained were fixed and stained with silver nitrate. **(D)** To quantify phosphate deposition after silver nitrate staining, the number of pixels per image (for a total of five images/donor) was counted by Image J. Images were acquired at 4× magnification. Scale bar represents 100 μm. Bars, ± standard deviation. ***P < 0.001 compared with vehicle-treated cells; ^#^ *P* < 0.05 compared with E2-treated cells; ^###^ *P* < 0.001 compared with E2-treated cells; ^ΨΨΨ^ *P* < 0.001 compared with daidzein-treated cells.

Due to the enhanced efficacy of analogs 2g and 2l, additional techniques were utilized to determine whether cells treated with 2g or 2l enhanced early osteogenesis. BMSCs and ASCs treated with vehicle, E2, daidzein, analog 2g, or analog 2l for 3 days were incubated in BCIP/NBT, the substrate for ALP. E2 enhanced BMSC and ASC differentiation by 2.0-fold and 1.6-fold, respectively; analog 2g enhanced differentiation by 2.1-fold and 2.4-fold; and analog 2l enhanced differentiation by 1.9-fold and 2.6-fold (*P* < 0.05; Figure 3A,B). While daidzein-treated BMSCs demonstrated enhanced ALP activity (1.6-fold increase; *P* < 0.05) relative to vehicle, daidzein-treated ASCs displayed similar ALP activity to vehicle-treated ASCs.

To assess phosphate deposition, which is secreted during late osteogenesis and is an essential component of the mature extracellular matrix of bone, cells were treated with vehicle, E2, daidzein, analog 2g, or analog 2l for 14 days and incubated in silver nitrate. While E2-treated and daidzein-treated BMSCs and ASCs demonstrated enhanced phosphate deposition, E2 treatment resulted in greater phosphate deposition than daidzein treatment (*P* < 0.001; Figure 3C,D). Structural modifications of daidzein into analogs 2g and 2l resulted in increased phosphate deposition (*P* < 0.001; Figure 3C,D). Treatment of BMSCs and ASCs with analog 2l resulted in the most significant increase in phosphate deposition compared with all other treatment groups (*P* < 0.001; Figure 3C,D).

Together, these results suggest that BMSCs and ASCs treated with E2 demonstrate enhanced osteogenesis through increased ALP activity and phosphate deposition. Daidzein analogs 2g and 2l also resulted in enhanced ALP activity and phosphate deposition. Treatment with daidzein was less effective than treatment with E2 or daidzein analogs.

### BMSCs and ASCs treated with 17β-estradiol demonstrate induction of early, middle, and late genes involved in osteogenesis

**Figure 4:**
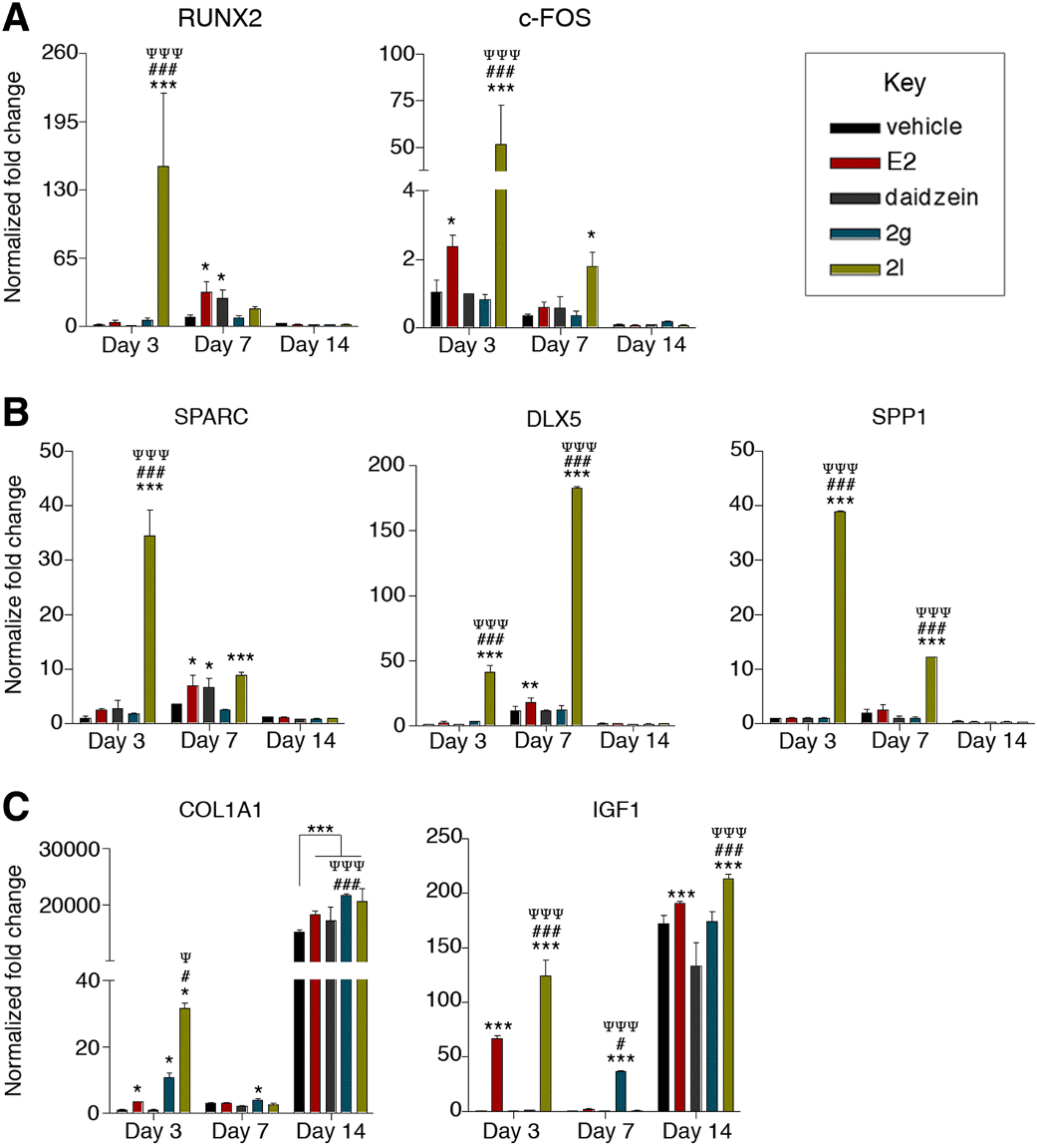
**Expression of early, middle, and late-stage lineage-specific osteogenic genes in bone marrow-derived mesenchymal stem cells was significantly induced by daidzein analogs.** Bone marrow-derived mesenchymal stem cells (BMSCs) were cultured in charcoal dextran-stripped osteogenic differentiation medium and concurrently treated with vehicle, 17β-estradiol (E2; 10 nM), daidzein (1 μM), or daidzein analog 2g or 2l (1 μM). Cells were collected after 3, 7, or 14 days of treatment. RNA was isolated from the cells and reverse transcribed into cDNA. Analysis of **(A)** early, **(B)** middle, or **(C)** late osteogenic transcription factors by quantitative polymerase chain reaction. Expression values are normalized to undifferentiated cells normalized to 1.0. Bars, ± standard deviation. *P < 0.05 compared to vehicle-treated BMSCs; ***P < 0.001 compared with vehicle-treated BMSCs; ^#^ *P* < 0.05 compared with E2-treated BMSCs; ^###^ *P* < 0.001 compared with E2-treated BMSCs; ^Ψ^ *P* < 0.05 compared with daidzein-treated BMSCs; ^ΨΨΨ^ *P* < 0.001 compared with daidzein-treated BMSCs.

**Figure 5:**
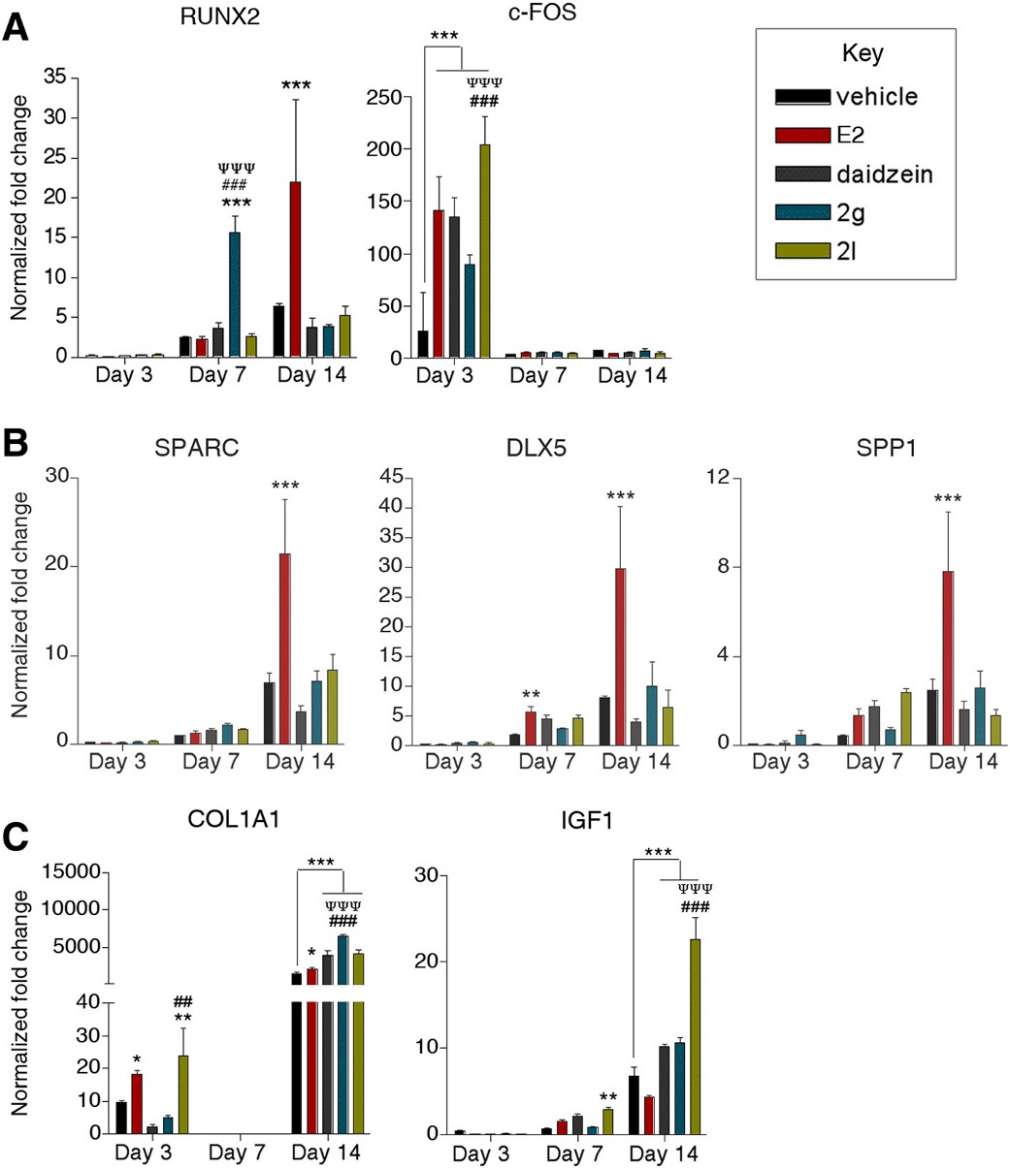
**Expression of early, middle, and late-stage lineage-specific osteogenic genes in adipose-derived stromal/stem cells was significantly induced by daidzein analogs.** Adipose-derived stromal/stem cells (ASCs) were cultured in charcoal dextran-stripped osteogenic differentiation medium and concurrently treated with vehicle, 17β-estradiol (E2; 10 nM), daidzein (1 μM), or daidzein analog 2g or 2l (1 μM). Cells were collected after 3, 7, or 14 days of treatment. RNA was isolated from the cells and reverse transcribed into cDNA. Analysis of **(A)** early, **(B)** middle, or **(C)** late osteogenic transcription factors by quantitative polymerase chain reaction. Expression values are normalized to undifferentiated cells normalized to 1.0. Bars, ± standard deviation. *P < 0.05 compared with vehicle-treated ASCs; **P < 0.01 compared with vehicle-treated ASCs; ***P < 0.001 compared with vehicle-treated ASCs; ^###^ *P* < 0.001 compared to E2-treated ASCs; ^ΨΨΨ^ *P* < 0.001 compared with daidzein-treated ASCs.

While BMSCs and ASCs treated with E2 displayed similar temporal induction of early and late osteogenic genes, significant differences were observed in the induction of middle osteogenic genes (Figures 4 and 5). More specifically, E2 induced the expression of early osteogenic genes (c-FOS and COL1A1) within 3 days in both BMSCs and ASCs (*P* < 0.05; Figures 4 and 5). However, the levels of gene induction varied between BMSCs and ASCs. Following 3 days of treatment, E2 increased c-FOS expression by 2.4-fold in BMSCs, while E2 enhanced c-FOS expression by 141.1-fold in ASCs, relative to undifferentiated cells. The induction of COL1A1 expression was different between BMSCs and ASCs: 3.4-fold in BMSCs and 18.3-fold in ASCs, relative to undifferentiated cells (*P* < 0.05; Figures 4 and 5). These results highlight the differences in response to E2 stimulation.

Furthermore, differences were observed in the effects of E2 on middle osteogenic genes both temporally and in relation to induction level. SPARC and DLX5 was upregulated following 7 days of E2 treatment in BMSCs, while SPARC, DLX5, and SPP1 was upregulated following 14 days of treatment in ASCs, relative to undifferentiated cells (*P* < 0.05; Figures 4 and 5). E2-treated BMSCs displayed higher levels of induction in the middle genes on day 7, relative to undifferentiated cells (SPARC, 6.9-fold in BMSCs vs. 1.3-fold in ASCs; DLX5, 17.8-fold in BMSCs vs. 5.8-fold in ASCs; Figures 4 and 5).

Additional differences between BMSCs and ASCs associated with E2 stimulation were in RUNX2 and IGF1 expression. RUNX2 expression in BMSCs was increased (9.1-fold) following 7 days of E2 treatment, while enhanced RUNX2 expression in ASCs (22.0-fold) did not occur until 14 days, relative to undifferentiated cells (*P* < 0.05; Figures 4 and 5). IGF1 induction was significantly enhanced in BMSCs following E2 treatment after 3 days in BMSCs, while this effect was not observed in ASCs (Figures 4 and 5; Additional file 5). Together, these results suggest that E2 stimulates the expression of osteogenic genes in both BMSCs and ASCs.

### Daidzein-treated, analog 2g-treated, and analog 2l-treated BMSCs and ASCs display different gene expression profiles

BMSCs and ASCs were treated with daidzein and collected after 3, 7, and 14 days. The expression of key osteogenic factors was investigated by quantitative polymerase chain reaction. Daidzein-treated BMSCs increased RUNX2 and SPARC expression by 9.1-fold and 3.6-fold, respectively, relative to undifferentiated BMSCs (*P* < 0.05; Figure 4). Daidzein-treated ASCs demonstrated increased c-FOS expression by 134.9-fold after 3 days, and COL1A1 and IGF1 expression by 3,899.6-fold and 10.2-fold, respectively, after 14 days, compared with undifferentiated ASCs (*P* < 0.001; Figure 5).

In contrast, BMSCs treated with analog 2g increased RUNX2 expression by 7.9-fold on day 7 and COL1A1 on days 3, 7, and 14 by 10.8-fold, 4.1-fold, and 21,600.4-fold, respectively (*P* < 0.05; Figure 4). The expression of COL1A1 was significantly more robust compared with E2-treated or daidzein-treated BMSCs on day 14 (*P* < 0.001; Figure 4C). ASCs treated with analog 2g demonstrated increased expression of COL1A1 compared with vehicle-treated, E2-treated, or daidzein-treated ASCs on day 14 (*P* < 0.001; Figure 5C).

Unlike E2, daidzein, or analog 2g, BMSCs treated with analog 2l showed increased expression of all early, middle, and late osteogenic genes investigated in this study. Relative to undifferentiated BMSCs, BMSCs treated with analog 2l demonstrated an enhanced expression of RUNX2, c-FOS, SPARC, DLX5, SPP1, COL1A1, and IGF1 by 152.8-fold, 51.6-fold, 34.4-fold, 41.2-fold, 38.8-fold, 31.8-fold, and 124.4-fold, respectively, on day 3 (*P* < 0.05; Figure 4). Daidzein analog 2l continued to induce the expression of c-FOS, SPARC, DLX5, and SPP1 by 1.8-fold, 8.9-fold, 182.8-fold, and 12.3-fold, respectively, on day 7 (*P* < 0.05; Figure 4). Additionally, analog 2l-treated BMSCs displayed increased late osteogenic genes COL1A1 and IGF1 even on day 14 (*P* < 0.05; Figure 4). These results suggest that analog 2l significantly induces osteogenesis through induction of key osteogenic regulatory genes to initiate and maintain osteogenesis.

While the effects of analog 2l on BMSCs were pronounced, the effects of analog 2l on ASCs were attenuated. ASCs treated with analog 2l displayed enhanced c-FOS expression by 204.5-fold after 3 days (*P* < 0.05; Figure 5). After 14 days of differentiation, analog 2l-treated ASCs displayed higher expression of COL1A1 (4,139.8-fold) compared with vehicle-treated (1,471.7-fold) or E2-treated (2,061.2-fold) ASCs (*P* < 0.05; Figure 5). Likewise, analog 2l-treated ASCs demonstrated enhanced IGF-1expression (22.6-fold) after 14 days, compared with vehicle-treated ASCs (6.8-fold) or E2-treated ASCs (4.4-fold; *P* < 0.05; Figure 5).

### Fulvestrant reversed the osteogenic effects of daidzein and analog 2g while only attenuating the effects of analog 2l

**Figure 6:**
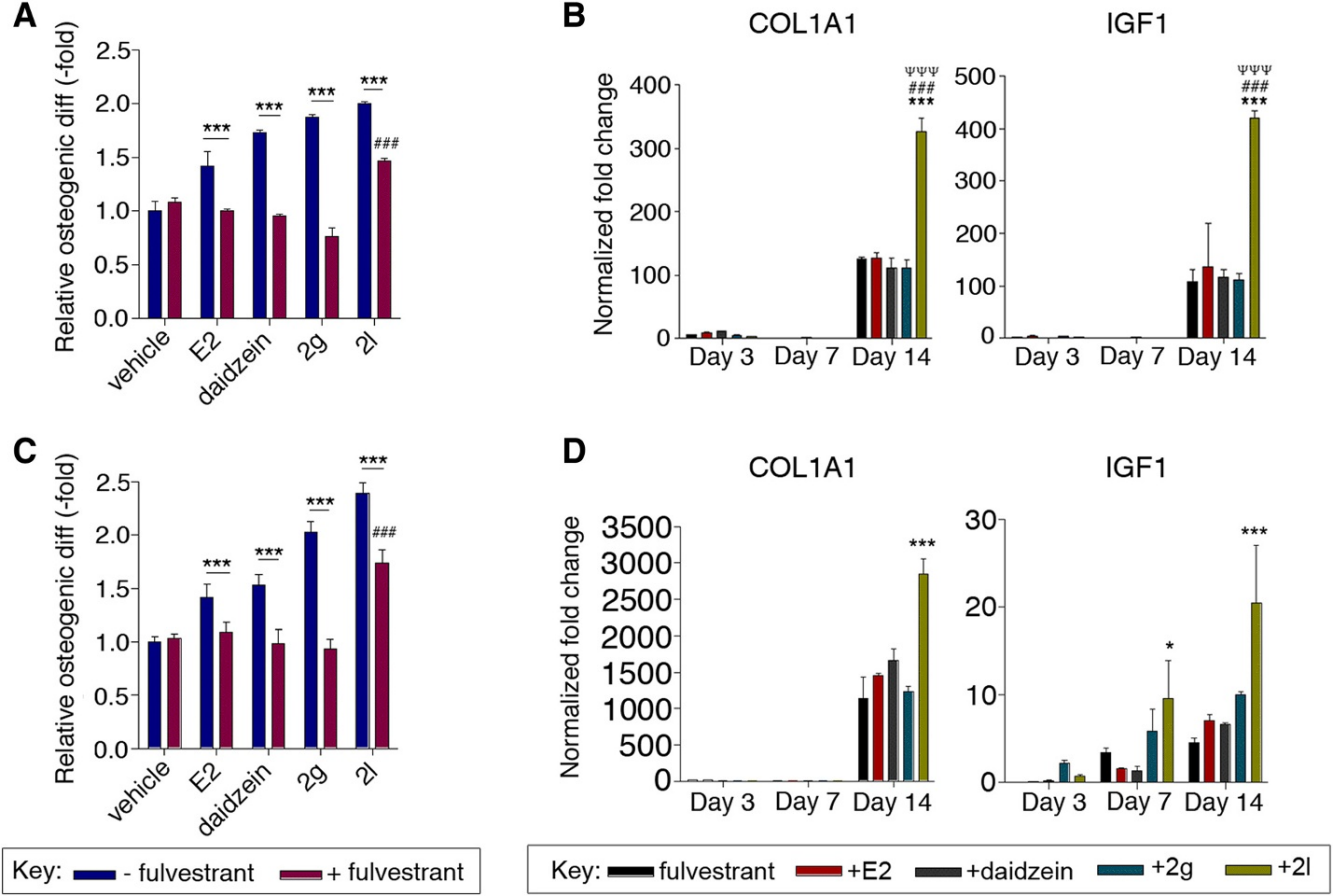
**Fulvestrant treatment reduces osteogenic induction by daidzein analogs through inhibition of early, middle, and/or late osteogenic transcription.** Bone marrow-derived mesenchymal stem cells (BMSCs) or adipose-derived stromal/stem cells (ASCs) were cultured in charcoal dextran-stripped osteogenic differentiation medium (CDS-ODM) and concurrently treated with vehicle, 17β-estradiol (E2; 10 nM), daidzein (1 μM), or daidzein analog 2g or 2l (1 μM) and fulvestrant. After 14 days, **(A)** BMSCs and **(C)** ASCs were stained with alizarin red and eluted with cetylpyridinium chloride (CPC). Quantification of the stain was measured at 590 nm and normalized to vehicle-treated cells. **(B)** BMSCs and **(D)** ASCs were collected after 3, 7, or 14 days of treatment. RNA was isolated from the cells and reverse transcribed into cDNA. Analyses of osteogenic transcription factors were assessed by quantitative polymerase chain reaction. Expression values are normalized to undifferentiated cells set to 1.0. Bars, ± standard deviation. *P < 0.05 compared with fulvestrant-treated cells; **P < 0.01 compared with fulvestrant-treated cells;***P < 0.001 compared with fulvestrant-treated cells; ^###^ *P* < 0.001 compared with fulvestrant and E2-treated cells; ^ΨΨΨ^ *P* < 0.001 compared with fulvestrant and daidzein-treated cells.

To determine the estrogenic activity of daidzein, ER inhibitor studies were performed on BMSCs and ASCs by treating cells simultaneously with daidzein and fulvestrant. Fulvestrant reduced the osteogenic potential of daidzein-treated BMSCs and ASCs from 1.7-fold to 1.0-fold and from 1.5-fold to 1.0-fold, respectively (Figure 6). While daidzein increased the expression of RUNX2 on day 7, simultaneous treatment with fulvestrant reduced RUNX2 expression by 56.0%, from 3.0-fold to 1.3-fold (*P* < 0.05; Figures 4 and 6; Additional files 6 and 7). With respect to ASCs, concomitant fulvestrant treatment reduced daidzein-induced c-FOS expression by 66.7% on day 3 (from 1.5-fold to 0.5-fold; *P* < 0.001), IGF1 by 85.7% on day 7 (from 2.8-fold to 0.4-fold; *P* < 0.01), and COL1A1 by 42.3% on day 14 (from 2.6-fold to 1.5-fold; *P* < 0.05; Figures 4 and 6; Additional files 5, 7 and 8). These inhibitor studies suggest that daidzein works through an ER-dependent mechanism as fulvestrant treatment reduced expression of genes activated by daidzein.

To investigate the activation of ER signaling pathways by analog 2g, inhibitor studies were conducted with BMSCs and ASCs by simultaneously treating cells with analog 2g and fulvestrant in CDS-ODM for 3, 7, or 14 days. Fulvestrant treatment reduced the osteogenic activity of analog 2g in BMSCs and ASCs to levels comparable with fulvestrant-treated cells from 1.9-fold to 0.8-fold and from 2.0-fold to 0.9-fold, respectively (*P* < 0.001; Figure 6). Furthermore, BMSCs treated with analog 2g and fulvestrant displayed reduced RUNX2 expression by 79.0% (from 4.3-fold to 0.9-fold) on day 3, COL1A1 expression by 91.6% (from 10.8-fold to 0.9-fold) on day 3, and IGF1 expression by 97.8% (from 40.9-fold to 0.9-fold) on day 7 (*P* < 0.05; Additional files 5, 6 and 7). With respect to ASCs, fulvestrant inhibited the activity of analog 2g by reducing RUNX2 expression on day 7 by 65.1% (from 6.3-fold to 2.2-fold) and COL1A1 expression on day 14 by 75% (from 4.4-fold to 1.1-fold; *P* < 0.01; Additional files 7, 8 and 9). Together, these results indicate that analog 2g acts through an ER-mediated mechanism, with reduced activity in the presence of fulvestrant.

Furthermore, to determine the estrogenic activity of analog 2l, concomitant treatment of 2l with fulvestrant resulted in reduced osteogenesis in BMSCs and ASCs. Osteogenic differentiation was not completely inhibited by fulvestrant, suggesting that analog 2l-induced osteogenic differentiation is through both ER-dependent and ER-independent pathways. Simultaneous delivery of fulvestrant with analog 2l reduced the osteogenesis of BMSCs (2.0-fold to 1.5-fold) and ASCs (2.4-fold to 1.7-fold; *P* < 0.001; Figure 6). Furthermore, fulvestrant reduced the expression of early and middle osteogenic genes in both BMSCs and ASCs, suggesting that the estrogenic mechanism of fulvestrant on osteogenesis is through the induction of early to middle osteogenic genes. COL1A1 and IGF1 expression remained induced in BMSCs even in the presence of fulvestrant (Figure 6; Additional files 2 and 9), suggesting that analog 2l utilizes an alternative non-ER-driven pathway to induce osteogenesis. Lastly, due to the impact of analog 2l on late osteogenic genes COL1A1 and IGF1, analysis of additional late osteogenic genes was conducted. Treatment with analog 2l increased DMP1 (3.9-fold) and SOST (2.2-fold) expression in BMSCs, while 2l had no effect on DMP1 or SOST expression in ASCs (Additional file 10). In BMSCs, fulvestrant treatment reversed the increased expression in SOST by analog 2l but did not alter DMP1 expression (Additional file 10). These data suggest that analog 2l selectively induces osteogenesis through non-ER-dependent mechanisms that result in the upregulation of COL1A1, IGF1, and SOST in BMSCs and of COLA1A and IGF1 in ASCs.

## Discussion

Osteoporosis is a disease characterized by decreasing BMD and a loss of the bone architecture, resulting in increased fragility and fracture incidence. To reduce the progression of the disease and increase bone strength, the development of new compounds to increase osteogenesis is necessary. In this study, synthetic daidzein analogs have been tested for their *in vitro* osteogenic potential in both BMSCs and ASCs. Daidzein analogs 2g and 2l were found to increase osteogenic differentiation characterized by alizarin red staining, ALP activity, and silver nitrate staining. In addition, these daidzein analogs enhanced osteogenic differentiation of BMSCs and ASCs relative to E2-treated or daidzein-treated cells. Furthermore, simultaneous treatment with fulvestrant eliminated the osteogenic activity of daidzein analog 2g and attenuated the osteogenic activity of analog 2l, suggesting that 2g acts predominantly through ER signaling while 2l may use both ER-dependent and ER-independent pathways. Analysis of transcript levels of key osteogenic genes showed that analogs 2g and 2l differentially induced osteogenic genes in BMSCs and ASCs. These results raise the potential that individual daidzein analogs may function through distinct ER signaling mechanisms such as ERα, ERβ or the G-protein-coupled ER. Recent research has demonstrated the involvement of G-protein-coupled ER as a mechanism of rapid ER signaling that can cross-talk with classic ER mechanisms or function in a distinct manner [25–27]. A combination of ERα/β-mediated and G-protein-coupled ER-mediated mechanisms may thus exist by which daidzein analogs influence the MSC and ASC differentiation responses. Evidence has also demonstrated that fulvestrant alone exhibits effects on gene expression apart from its anti-estrogenic effects, which further supports the possibility that certain daidzein analogs may function through distinct G-protein-coupled ER-dependent or ER-independent pathways [28–30].

Consistent with previously published studies, genistein and daidzein increased the osteogenic potential of BMSCs and ASCs. Previous work by Bitto and colleagues demonstrated that genistein enhanced the BMD but also restored structure to ovariectomy-induced osteoporotic bone in rats [31, 32]. Furthermore, the effects of genistein treatment in rats improved the overall architecture and strength of the bone better than raloxifene, a commonly used selective ER modulator used to treat osteoporosis [31, 32]. Comparative studies have shown that daidzein is more effective than genistein in preventing ovariectomy-induced bone loss in rats [33]. Indeed, daidzein was shown to enhance BMD in lumbar vertebrae, femur, and in the metaphyseal and diaphyseal zones, which have been shown to be rich in cancellous and cortical bone, respectively [33]. Daidzein treatment has also been shown to increase biomechanical strength by increasing collagen formation, while reducing osteoclast activity to limit the amount of degradation to the extracellular matrix [34, 35]. Together, daidzein treatment leads to reduced resorptive activity and increased anabolic activity in bone. The results of this study provide additional support for the anabolic activity of daidzein in BMSCs and ASCs. Additional studies have shown that daidzein with high calcium preserves bone mass and biomechanical strength in multiple sites in an ovariectomized mouse model [36], providing for the supplementation of daidzein with current osteoporosis treatment regimes.

While these phytoestrogens have proven effective in increasing bone density in rodent models, novel daidzein derivatives developed by our group were tested on BMSCs and ASCs to determine their potential to enhance bone differentiation and regeneration. Studies have shown that derivatives of genistein and daidzein have yielded better outcomes as anti-osteoporotic compounds compared with their original forms, either increasing anabolic activity or decreasing resorption activity. Wang and colleagues demonstrated that genistein derivatives act as potential selective ER modulators and increased the weight of bone in the femur relative to no treatment or treatment with genistein [37]. Other soy derivatives have been shown to increase *in vitro* osteoblast maturation in primary cultures of rat calvarial osteoblasts, to stimulate the differentiation of osteoblasts, and to increase the transcript levels of osteogenic genes involved in differentiation and mineralization [38]. Yadav and colleagues reported that modifying the two hydroxyl groups into alkoxy groups could lead to synthetic daidzein derivatives with altered potency [39]. One such compound, 7-(2-diethylamino-ethoxy)-3-(4-methoxy-phenyl)-4H-chromen-4-one, increased mineralization of bone marrow osteoprogenitor cells and increased mRNA expression of bone morphogenetic protein-2 and osteocalcin [39]. Our approach only modified the 7-hydroxy moiety by substituting the hydrogen with an isopropyl (daidzein analog 2c), a cyclopentyl (daidzein analog 2g), or an allyl (daidzein analog 2l) while retaining the 4-hydroxy moiety, rather than modifying both hydroxyl groups. We have previously studied the effect of such structural modifications on the estrogenic activity of daidzein analogs and demonstrated the sensitivity of 7-hydroxy substitution to the agonist/antagonist propensity of the daidzein derivatives [21]. While all three analogs have lower estrogenic activity than daidzein [21, 22], the specific alkyl substitution of the 7-hydroxy hydrogen yielded significantly increased osteogenic activity. Higher dosages of compounds 2g and 2l in our study did not negatively impact the osteogenic activity of the cells, nor lead to cytotoxicity. Additional studies of structure–activity relationships are underway in our laboratories to determine whether further structural alterations at the other sites will provide increased potency and/or maintain the enhanced efficacy that has been gained by modifications of the 7-hydroxy moiety.

Furthermore, previous studies have also attributed the osteogenic effects of daidzein to the production of equol in the gut. Our previous study thus focused on structurally modifying daidzein to generate equol analogs and investigated whether the equol structural motif conferred greater osteogenic potency [20]. However, our results suggested that the daidzein analogs investigated in this paper, 2g and 2l, demonstrated greater osteogenic activity than the equol analogs. Additional equol analogs are being prepared in our laboratory, which may provide more definitive evidence for enhance osteogenic activity compared with the equol analogs investigated previously.

Although analog 2g possessed much weaker estrogenic activity than E2, 2g treatment enhanced RUNX2, SPARC, and IGF1 in BMSCs and enhanced RUNX2 and COL1A1 in ASCs. RUNX2 is essential for osteoblast development and proper bone formation, regulating transcription of numerous genes that control osteoblast development from mesenchymal stem cells and maturation [40, 41]. SPARC, IGF1, and COL1A1 have all been implicated in increasing BMD, increasing biomechanical strength, and maintaining the extracellular matrix [42]. The inhibitory effect of fulvestrant on the osteogenic activity of analog 2g-treated BMSCs and ASCs provide additional support for the estrogenic activity of 2g, suggesting that the weaker analog 2g appears to enhance osteogenic activity through signaling pathways associated with ER.

Likewise, structural modification of daidzein into analog 2l also enhanced osteogenic differentiation of BMSCs and ASCs, possibly through ER pathways. Interestingly, unlike analog 2g, fulvestrant treatment did not abolish the osteogenic activity of analog 2l, suggesting that 2l is likely to be acting through an ER-independent mechanism. To confirm this finding, the downstream effect of analog 2l on BMSCs and ASCs were explored in this study. The molecule targeted by analog 2l will probably converge with the identification of key downstream osteogenic genes identified here. BMSCs and ASCs treated with analog 2l demonstrated alterations in the transcriptional level of select early, middle, and late osteogenic genes involved in differentiation of the cells and mineralization of the extracellular matrix. In BMSCs treated with analog 2l, all osteogenic genes were upregulated, suggesting a powerful effect of 2l on BMSCs. In contrast, ASCs treated with analog 2l demonstrated a less pronounced effect, altering only c-FOS, COL1A1, and IGF1 [43].

While our study focused on osteogenic activity of the daidzein analogs, it should be noted that the daidzein analogs reduced adipogenic differentiation at 1 μM concentration. While our treatment of genistein and daidzein did not yield significant reduction in BMSCs and ASC adipogenic differentiation, it is possible that the dosage used was not adequate to see an effect. Kim and colleagues determined that a 20 μM concentration was necessary to reduce adipogenic differentiation of ASCs, and higher concentrations resulted in greater inhibition [44]. Delivery of daidzein also reduced body weight in obesity-induced animals and reduced the expression of adipogenic genes in a dose-dependent manner [44–46]. Additional studies have shown that daidzein derivatives have likewise been effective in treating high-fat diet-induced male obese mice [47]. These previous studies, in conjunction with our current study, would suggest further investigating the use of daidzein analogs to reduce diet-induced obesity, as these analogs were more potent than daidzein.

To our knowledge, this study is the first to examine the effects of genistein or daidzein on bone differentiation in human ASCs. Our study demonstrated that these compounds have similar effects on ASCs as on BMSCs. Our study of daidzein analogs further shows that minor structural modifications of daidzein further increased the osteogenic differentiation of BMSCs and ASCs, although the effect was more pronounced in ASCs. Both BMSCs and ASCs are derived from the same germ layer and as such possess similar biologic characteristics. However, in-depth analysis of these two cell types has recently revealed differences in immunophenotypical and gene expression profiles [48–50]. Monaco and colleagues determined that ASCs have larger lipid metabolism, migration, and immunomodulatory capacity during early osteogenic differentiation compared with BMSCs, while BMSCs have larger induction of inflammation, cell growth, and proliferation [48]. Consistent with previous reports, our study suggests that differences exist between BMSCs and ASCs which may account for the differences in the transcript levels of osteogenic genes following induction by daidzein analogs. More specifically, BMSCs and ASCs treated with the same daidzein analog express different mRNA levels of osteogenic factors. Furthermore, recent studies have combined the availability of ASCs and their osteogenic differentiation for tissue engineering [51]. ASCs and BMSCs have been seeded onto extracellular matrix scaffolds to increase bone formation [52]. The daidzein analogs investigated in this study could be coated onto these scaffolds to determine their *in vivo* efficacy in future experiments.

Lastly, as these compounds would be used in postmenopausal women, it is important for these compounds to have no effect on breast cancer and endometrial cancer. Theoretically, a drug used to treat osteoporosis should increase BMD without increasing the risk of cancer. An important advantage of the daidzein analogs compounds is the limited estrogenic effect of these compounds on cancer cells. Previous studies have determined that daidzein and daidzein derivatives have negligible effects on cancer growth or progression [21, 53–57]. As such, it is possible that these compounds could be used in combination with chemotherapy or other forms of cancer therapy to reduce the incidence of osteoporosis in at-risk patients. Furthermore, these compounds could be used in combination with other anti-osteoporotic drugs to reduce osteoporosis [58]. However, additional *in vivo* analyses of these compounds in osteoporosis-induced ovariectomized mice or rats or in critical-sized calvarial defect models are necessary to determine the regenerative potential of these compounds as well as to determine the pharmacokinetics of these compounds for potential combination therapy.

## Conclusion

Osteoporosis is a debilitating disease associated with reduced BMD, increased incidence of fractures, poor mobility, and increased morbidity and mortality. While current therapeutic interventions have been focused on anti-resorptive drugs, development of anabolic drugs that increase bone regeneration is necessary to compensate for bone loss during osteoporosis. In this study, the use of synthetic daidzein derivatives was investigated for the induction of *in vitro* osteogenesis in BMSCs and ASCs and provided potential mechanisms of action of these compounds. While these data provide a foundation for further analyses of the daidzein analogs in BMSCs or ASCs, *in vitro* or *in vivo*, future studies are necessary to investigate the role of these compounds in a rodent ovariectomized model of osteoporosis on BMD and bone architecture and for the translation of these compounds clinically to improve the outcomes of patients diagnosed with osteoporosis.

## Electronic supplementary material

Additional file 1: **Shows a characterization of BMSCs and ASCs.** BMSCs (*n* = 6) and ASCs (*n* = 6) were each induced to undergo osteogenic and adipogenic differentiation, immunophenotyped for cell surface antigens with flow cytometry, and the colony formation potential determined. (A) BMSCs and ASCs were cultured in ODM or ADM for 14 days and stained with alizarin red or oil red O, respectively. Representative images of osteogenesis (4× magnification) and adipogenesis (10× magnification) in BMSCs and ASCs are shown. Scale bar represents 200 μm. (B) Cells were stained with cell surface markers, CD44, CD90, CD106, CD166, CD11b, CD34, and CD45 and their respective isotype controls. Each overlay contains the isotype control for each cell type and the cell surface marker of interest. (C) BMSCs and ASCs were plated at 100 cells per 10 cm plate and were stained with crystal violet after 14 days in culture to visualize the colony-forming units. (TIFF 4 MB)

Additional file 2: **Shows daidzein analogs inhibit adipogenesis.** BMSCs (*n* = 6) and ASCs (*n* = 6) were each induced to undergo adipogenic differentiation and treated with vehicle, E2, daidzein, or daidzein analog (1 μM) for 14 days and stained with oil red O. (A) Representative images of cells stained with oil red O are shown at 10× magnification. Scale bar represents 100 μm. (B) To quantify the amount of oil red O staining in treated BMSCs and ASCs, cells were eluted with isopropanol and measured at 544 nm. Adipogenic differentiation was determined relative to vehicle-treated cells (normalized to 1.0). Bars, ± standard deviation. *P < 0.05; **P < 0.01; ***P < 0.001. (TIFF 3 MB)

Additional file 3: **Shows analogs 2g and 2l do not demonstrate cytotoxic or proliferative effects on BMSCs and ASCs.** (A) BMSCs (*n* = 6) and (B) ASCs (*n* = 6) were each cultured in CCM supplemented with vehicle or daidzein analog (1 μM) for 7 or 14 days and assessed by MTT assay. Bars, ± standard deviation. (TIFF 2 MB)

Additional file 4: **Presents the EC**_**50**_ **of each compound in BMSCs and ASCs.** Values presented in molar (M) concentration. (DOC 30 KB)

Additional file 5: **Presents the gene expression profile of BMSCs and ASCs treated with E2, daidzein, analog 2g, analog or 2l in the presence and absence of fulvestrant on day 3.** Data normalized to vehicle-treated cells after 3 days. *P < 0.05; **P < 0.01; ***P < 0.001 relative to the respective vehicle-treated cells. (DOC 51 KB)

Additional file 6: **Shows that ER antagonist fulvestrant inhibits the expression of osteogenic genes induced by daidzein analogs in BMSCs.** BMSCs were cultured in CDS-ODM and concurrently treated with vehicle, E2 (10 nM), daidzein (1 μM), or daidzein analog (1 μM) and ER antagonist fulvestrant. Cells were collected after 3, 7, or 14 days of treatment. RNA was isolated from the cells and reverse transcribed into cDNA. Analyses of osteogenic genes were assessed by quantitative polymerase chain reaction. Expression values are normalized to undifferentiated cells, normalized to 1.0. Bars, ± standard deviation. (TIFF 2 MB)

Additional file 7: **Presents the gene expression profile of BMSCs and ASCs treated with E2, daidzein, analog 2g, or analog 2l in the presence and absence of fulvestrant on day 7.** Data normalized to vehicle-treated cells after 7 days. *P < 0.05; **P < 0.01; ***P < 0.001 relative to the respective vehicle-treated cells. (DOC 51 KB)

Additional file 8: **Presents the gene expression profile of BMSCs and ASCs treated with E2, daidzein, analog 2g, or analog 2l in the presence and absence of fulvestrant on day 14.** Data normalized to vehicle-treated cells after 14 days. *P < 0.05; **P < 0.01; ***P < 0.001 relative to the respective vehicle-treated cells. (DOC 51 KB)

Additional file 9: **Shows that fulvestrant-treated ASCs demonstrate reduced expression of osteogenic transcription factors induced by daidzein analogs.** ASCs were cultured in CDS-ODM and concurrently treated with vehicle, E2 (10 nM), daidzein (1 μM), or daidzein analog (1 μM) and ER antagonist fulvestrant (100 nM). Cells were collected after 3, 7, or 14 days of treatment. RNA was isolated from the cells and reverse transcribed into cDNA. Analyses of osteogenic genes were assessed by quantitative polymerase chain reaction. Expression values are normalized to undifferentiated vehicle-treated cells, normalized to 1.0. Bars, ± standard deviation. *P < 0.05; **P < 0.01; ***P < 0.001 relative to vehicle-treated cells. (TIFF 2 MB)

Additional file 10: **Presents the gene expression profile of BMSCs and ASCs treated with analog 2l in the presence and absence of fulvestrant on day 14.** Data normalized to vehicle-treated cells after 14 days. *P < 0.05; relative to the respective vehicle-treated cells. (DOC 31 KB)

## Abbreviations

ALP: alkaline phosphatase
ASC: adipose-derived stromal/stem cell
BCIP/NBT: 5-bromo-4-chloro-3-indolyl phosphate
BMD: bone mineral density
BMSC: bone marrow-derived mesenchymal stem cell
CCM: complete culture media
CDS-ADM: charcoal dextran-stripped adipogenic differentiation medium
CDS-ODM: charcoal dextran-stripped osteogenic differentiation medium
c-FOS: FBJ murine osteosarcoma viral oncogene homolog
CO2: carbon dioxide
COL1A1: collagen type 1 alpha
DLX5: distal-less homeobox 5
DMP1: dentin matrix acidic phosphoprotein 1
E2: 17β-estradiol
EC50: half-maximal effective concentration
EDTA: ethylenediamine tetraacetic acid
ER: estrogen receptor
IGF1: insulin-like growth factor 1
ODM: osteogenic differentiation medium
PBS: phosphate-buffered saline
RUNX2: runt-related transcription factor 2
SOST: sclerostin
SPARC: osteonectin
SPP1: secreted phosphoprotein 1.
