# Supplementary figures and images for: Novel daidzein analogs enhance osteogenic activity of bone marrow-derived mesenchymal stem cells and adipose-derived stromal/stem cells through estrogen receptor dependent and independent mechanisms
Source: Stem Cell Res Ther. 2014 Aug 28;5(4):105. doi: 10.1186/scrt493 (PMC4355363; doi:10.1186/scrt493)

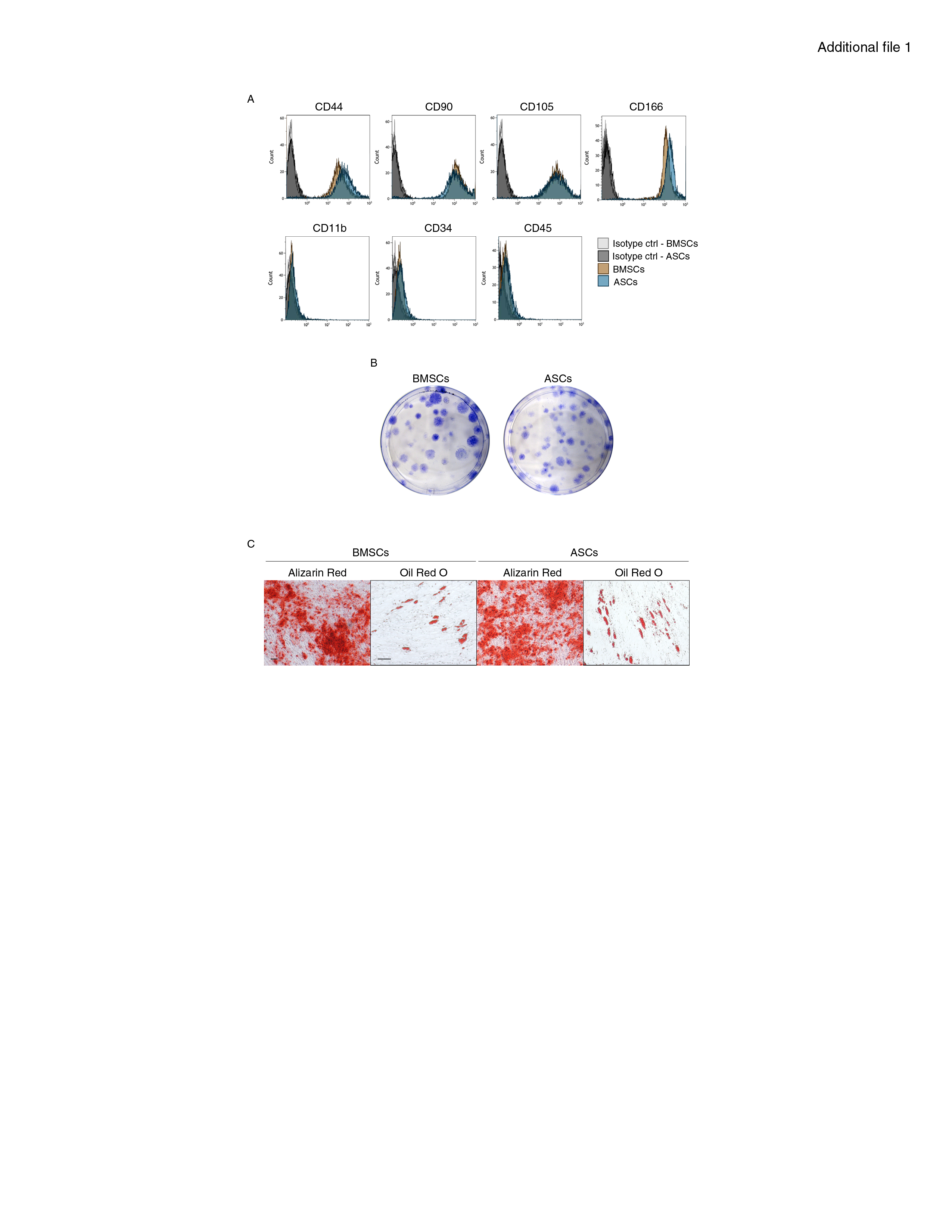

Supplement: Supplementary file 1 — Additional file 1: Shows a characterization of BMSCs and ASCs. BMSCs (n = 6) and ASCs (n = 6) were each induced to undergo osteogenic and adipogenic differentiation, immunophenotyped for cell surface antigens with flow cytometry, and the colony formation potential determined. (A) BMSCs and ASCs were cultured in ODM or ADM for 14 days and stained with alizarin red or oil red O, respectively. Representative images of osteogenesis (4× magnification) and adipogenesis (10× magnification) in BMSCs and ASCs are shown. Scale bar represents 200 μm. (B) Cells were stained with cell surface markers, CD44, CD90, CD106, CD166, CD11b, CD34, and CD45 and their respective isotype controls. Each overlay contains the isotype control for each cell type and the cell surface marker of interest. (C) BMSCs and ASCs were plated at 100 cells per 10 cm plate and were stained with crystal violet after 14 days in culture to visualize the colony-forming units. (TIFF 4 MB) [file 13287_2014_413_MOESM1_ESM.tiff]

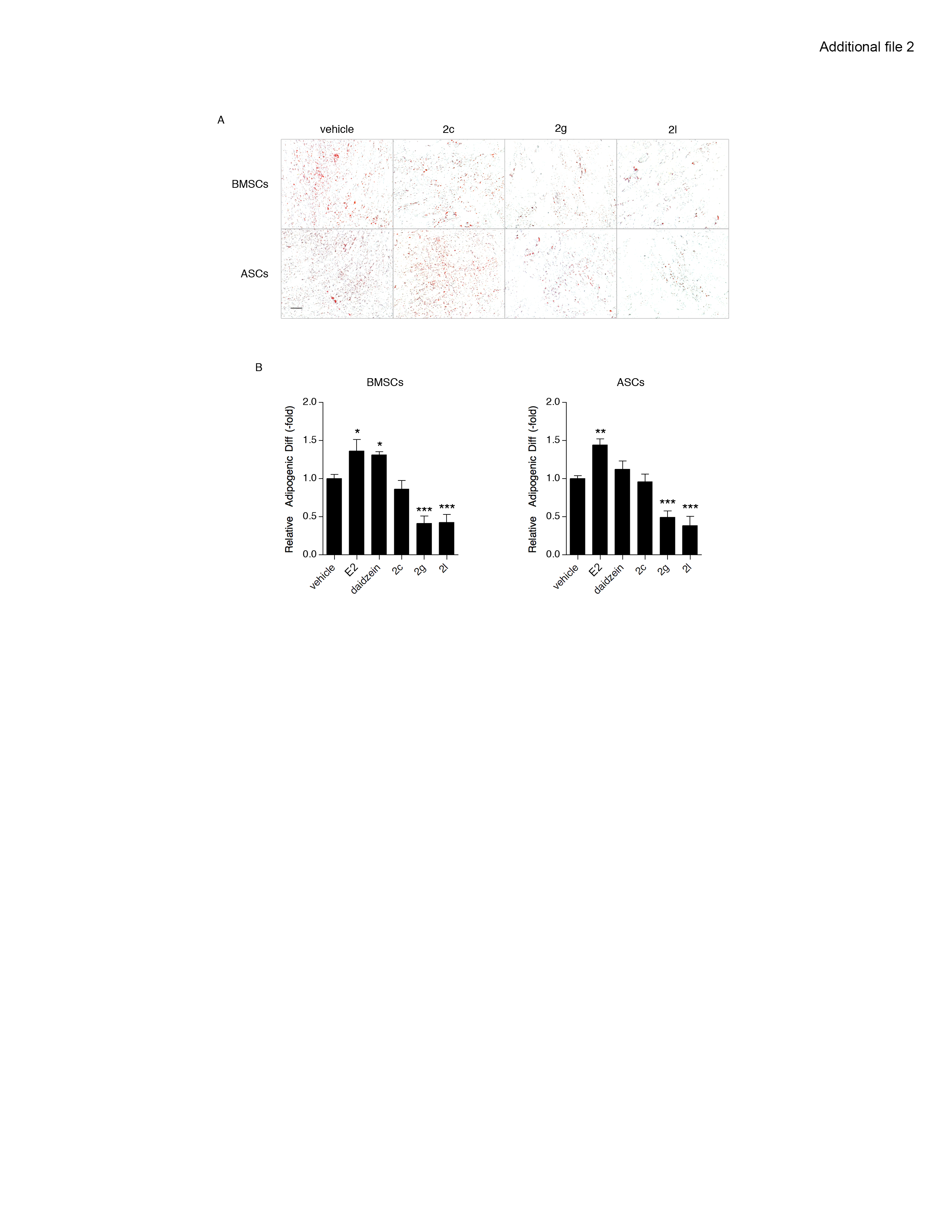

Supplement: Supplementary file 2 — Additional file 2: Shows daidzein analogs inhibit adipogenesis. BMSCs (n = 6) and ASCs (n = 6) were each induced to undergo adipogenic differentiation and treated with vehicle, E2, daidzein, or daidzein analog (1 μM) for 14 days and stained with oil red O. (A) Representative images of cells stained with oil red O are shown at 10× magnification. Scale bar represents 100 μm. (B) To quantify the amount of oil red O staining in treated BMSCs and ASCs, cells were eluted with isopropanol and measured at 544 nm. Adipogenic differentiation was determined relative to vehicle-treated cells (normalized to 1.0). Bars, ± standard deviation. *P < 0.05; **P < 0.01; ***P < 0.001. (TIFF 3 MB) [file 13287_2014_413_MOESM2_ESM.tiff]

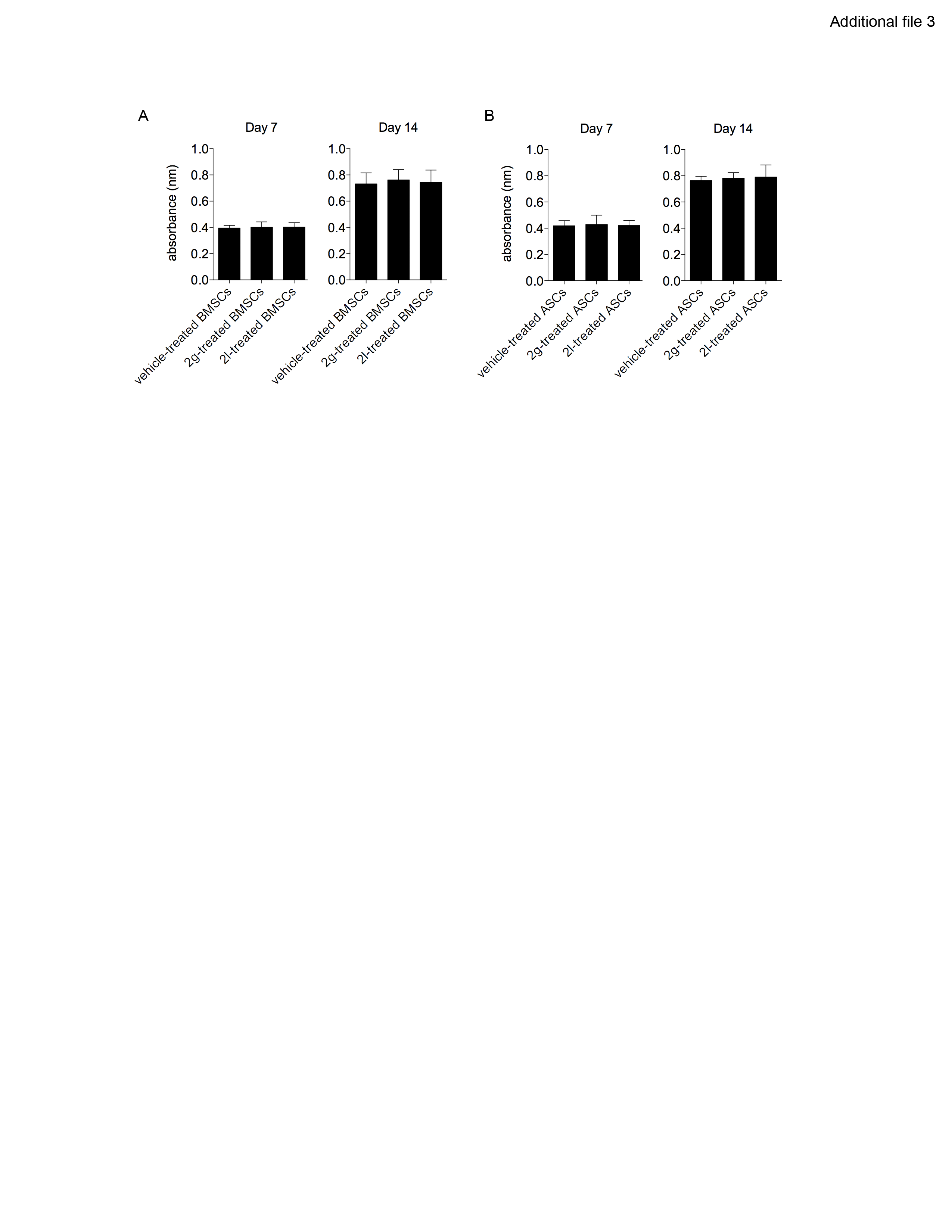

Supplement: Supplementary file 3 — Additional file 3: Shows analogs 2g and 2l do not demonstrate cytotoxic or proliferative effects on BMSCs and ASCs. (A) BMSCs (n = 6) and (B) ASCs (n = 6) were each cultured in CCM supplemented with vehicle or daidzein analog (1 μM) for 7 or 14 days and assessed by MTT assay. Bars, ± standard deviation. (TIFF 2 MB) [file 13287_2014_413_MOESM3_ESM.tiff]

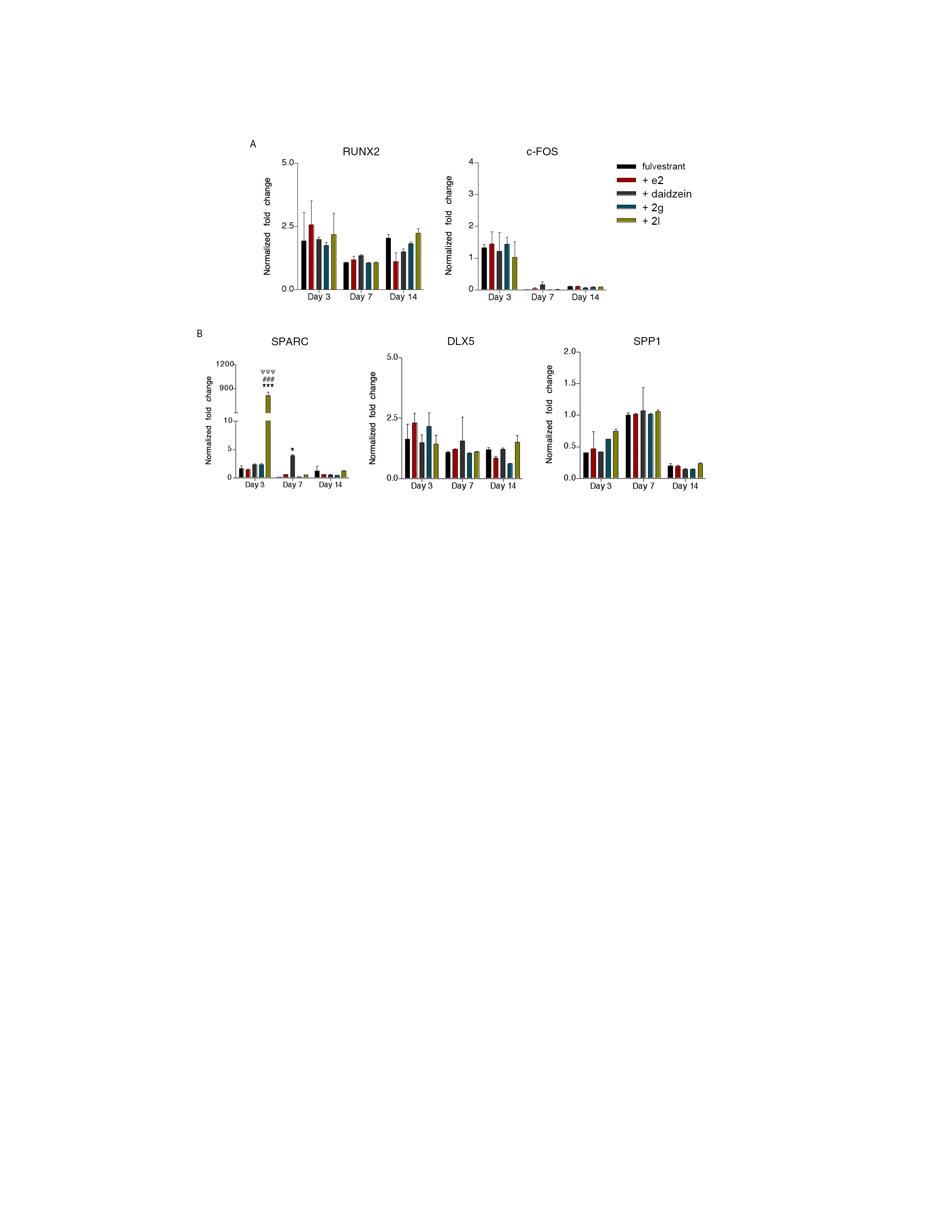

Supplement: Supplementary file 6 — Additional file 6: Shows that ER antagonist fulvestrant inhibits the expression of osteogenic genes induced by daidzein analogs in BMSCs. BMSCs were cultured in CDS-ODM and concurrently treated with vehicle, E2 (10 nM), daidzein (1 μM), or daidzein analog (1 μM) and ER antagonist fulvestrant. Cells were collected after 3, 7, or 14 days of treatment. RNA was isolated from the cells and reverse transcribed into cDNA. Analyses of osteogenic genes were assessed by quantitative polymerase chain reaction. Expression values are normalized to undifferentiated cells, normalized to 1.0. Bars, ± standard deviation. (TIFF 2 MB) [file 13287_2014_413_MOESM6_ESM.tiff]

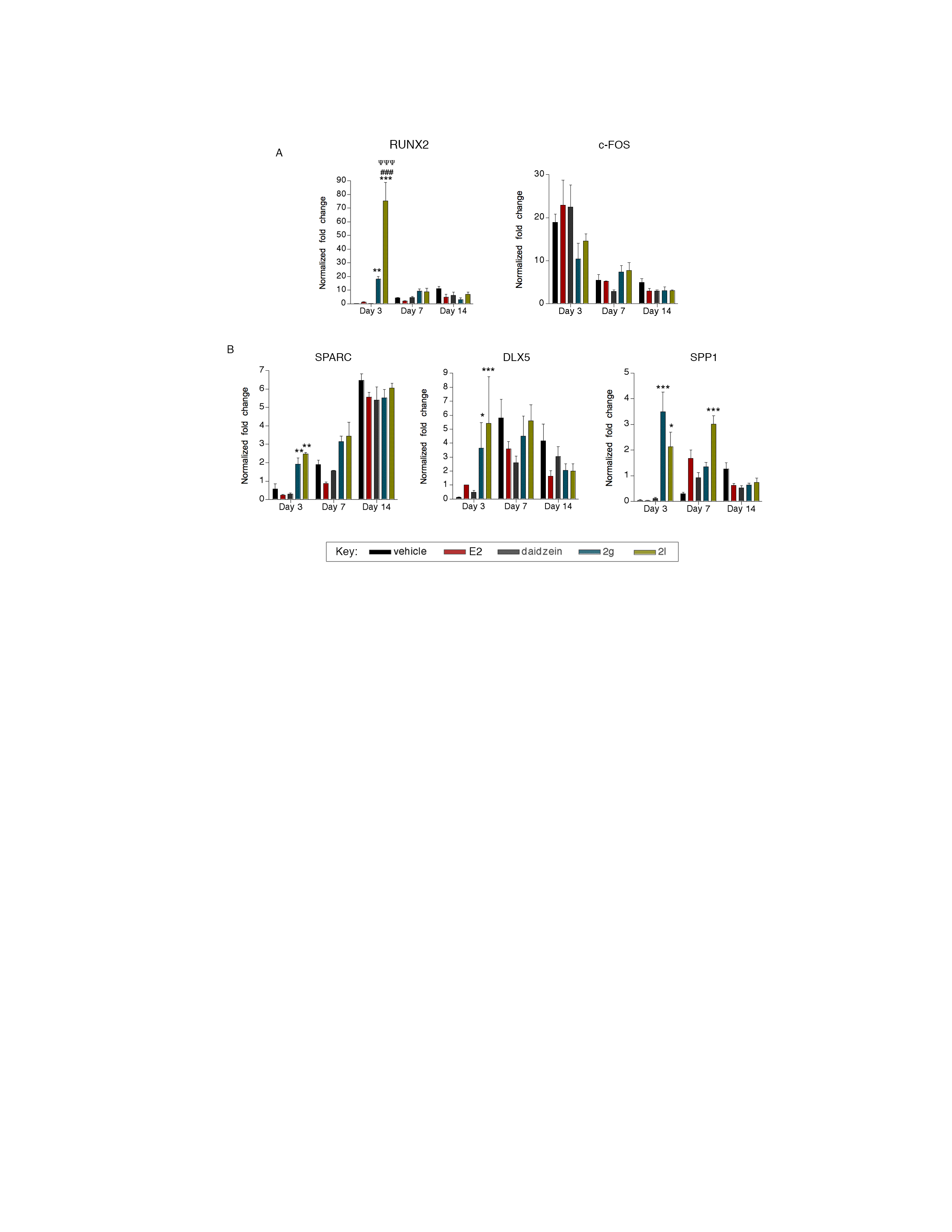

Supplement: Supplementary file 9 — Additional file 9: Shows that fulvestrant-treated ASCs demonstrate reduced expression of osteogenic transcription factors induced by daidzein analogs. ASCs were cultured in CDS-ODM and concurrently treated with vehicle, E2 (10 nM), daidzein (1 μM), or daidzein analog (1 μM) and ER antagonist fulvestrant (100 nM). Cells were collected after 3, 7, or 14 days of treatment. RNA was isolated from the cells and reverse transcribed into cDNA. Analyses of osteogenic genes were assessed by quantitative polymerase chain reaction. Expression values are normalized to undifferentiated vehicle-treated cells, normalized to 1.0. Bars, ± standard deviation. *P < 0.05; **P < 0.01; ***P < 0.001 relative to vehicle-treated cells. (TIFF 2 MB) [file 13287_2014_413_MOESM9_ESM.tiff]
