# Supplementary material for: Novel daidzein analogs enhance osteogenic activity of bone marrow-derived mesenchymal stem cells and adipose-derived stromal/stem cells through estrogen receptor dependent and independent mechanisms
Source: Stem Cell Res Ther. 2014 Aug 28;5(4):105. doi: 10.1186/scrt493 (PMC4355363; doi:10.1186/scrt493)
Supplement: Supplementary file 4 — Additional file 4: Presents the EC 50 of each compound in BMSCs and ASCs. Values presented in molar (M) concentration. (DOC 30 KB) [file 13287_2014_413_MOESM4_ESM.doc]

| Treatment | BMSCs | ASCs |
| --- | --- | --- |
| E2 | -8.35 1 0.13 | -9.20 ± 0.12 |
| Daidzein | -6.62 ± 0.10 | -7.76 ± 0.10 |
| Daidzein analog 2g | -6.99 ± 0.20 | -7.53 ± 0.15 |
| Daidzein analog 2l | -6.99 ± 0.22 | -7.32 ± 0.09 |
