# Supplementary material for: Novel daidzein analogs enhance osteogenic activity of bone marrow-derived mesenchymal stem cells and adipose-derived stromal/stem cells through estrogen receptor dependent and independent mechanisms
Source: Stem Cell Res Ther. 2014 Aug 28;5(4):105. doi: 10.1186/scrt493 (PMC4355363; doi:10.1186/scrt493)
Supplement: Supplementary file 8 — Additional file 8: Presents the gene expression profile of BMSCs and ASCs treated with E2, daidzein, analog 2g, or analog 2l in the presence and absence of fulvestrant on day 14. Data normalized to vehicle-treated cells after 14 days. *P < 0.05; **P < 0.01; ***P < 0.001 relative to the respective vehicle-treated cells. (DOC 51 KB) [file 13287_2014_413_MOESM8_ESM.doc]

| Gene Name | BMSCs | | ASCs | |
| --- | --- | --- | --- | --- |
|  | - Fulvestrant | + Fulvestrant | - Fulvestrant | + Fulvestrant |
|  |  |  |  |  |
| **E2** | | | | |
| RUNX2 | 0.7 ± 0.0 | 0.5 ± 0.2 | 3.4 ± 1.6 | 0.4 ± 0.2* |
| c-FOS | 0.8 ± 0.1 | 1.0 ± 0.2 | 0.6 ± 0.0 | 0.6 ± 0.1 |
| SPARC | 0.9 ± 0.1 | 0.5 ± 0.0** | 3.1 ± 0.9 | 0.9 ± 0.0* |
| DLX5 | 0.8 ± 0.1 | 0.7 ± 0.1 | 3.7 ± 1.3 | 0.4 ± 0.1* |
| SPP1 | 0.6 ± 0.2 | 1.0 ± 0.1 | 3.1 ± 1.0 | 0.5 ± 0.1* |
| COL1A1 | 1.2 ± 0.0 | 1.0 ± 0.1 | 1.4 ± 0.1 | 1.3 ± 0.0 |
| IGF1 | 1.1 ± 0.0 | 1.3 ± 0.8 | 0.6 ± 0.0 | 1.5 ± 0.2*** |
|  |  |  |  |  |
|  |  |  |  |  |
| **daidzein** | | | | |
| RUNX2 | 0.6 ± 0.0 | 0.7 ± 0.1 | 0.6 ± 0.2 | 0.5 ± 0.2 |
| c-FOS | 1.0 ± 0.0 | 0.6 ± 0.1** | 0.7 ± 0.1 | 0.6 ± 0.1 |
| SPARC | 0.7 ± 0.0 | 0.4 ± 0.1** | 0.5 ± 0.1 | 0.9 ± 0.1 |
| DLX5 | 0.7 ± 0.1 | 1.0 ± 0.0 | 0.5 ± 0.1 | 0.7 ± 0.2 |
| SPP1 | 0.6 ± 0.0 | 0.7 ± 0.1 | 0.6 ± 0.1 | 0.4 ± 0.1 |
| COL1A1 | 1.1 ± 0.2 | 0.9 ± 0.1 | 2.6 ± 0.5 | 1.5 ± 0.1* |
| IGF1 | 0.8 ± 0.1 | 1.1 ± 0.1 | 1.5 ± 0.0 | 1.5 ± 0.0 |
|  |  |  |  |  |
|  |  |  |  |  |
| **2g** | | | | |
| RUNX2 | 0.5 ± 0.0 | 0.9 ± 0.0 | 0.6 ± 0.0 | 0.3 ± 0.1 |
| c-FOS | 1.8 ± 0.3 | 0.8 ± 0.1** | 0.9 ± 0.2 | 0.6 ± 0.2 |
| SPARC | 0.7 ± 0.1 | 0.4 ± 0.0** | 1.0 ± 0.2 | 0.9 ± 0.1 |
| DLX5 | 0.8 ± 0.0 | 0.5 ± 0.0*** | 1.2 ± 0.5 | 0.5 ± 0.1 |
| SPP1 | 0.7 ± 0.2 | 0.7 ± 0.1 | 1.0 ± 0.3 | 0.5 ± 0.1* |
| COL1A1 | 1.4 ± 0.0 | 0.9 ± 0.1*** | 4.4 ± 0.1 | 1.1 ± 0.1**** |
| IGF1 | 1.0 ± 0.1 | 1.0 ± 0.1 | 1.6 ± 0.1 | 2.2 ± 0.1*** |
|  |  |  |  |  |
|  |  |  |  |  |
| **2l** | | | | |
| RUNX2 | 0.7 ± 0.0 | 1.1 ± 0.1 | 0.8 ± 0.2 | 0.6 ± 0.1 |
| c-FOS | 0.8 ± 0.1 | 0.9 ± 0.0 | 0.6 ± 0.2 | 0.6 ± 0.0 |
| SPARC | 0.8 ± 0.0 | 1.0 ± 0.1 | 1.2 ± 0.3 | 0.9 ± 0.0 |
| DLX5 | 0.8 ± 0.0 | 1.3 ± 0.2 | 0.8 ± 0.4 | 0.5 ± 0.1 |
| SPP1 | 0.5 ± 0.0 | 1.2 ± 0.1*** | 0.5 ± 0.1 | 0.6 ± 0.1 |
| COL1A1 | 1.4 ± 0.2 | 2.6 ± 0.2 | 2.8 ± 0.3 | 2.5 ± 0.2 |
| IGF1 | 1.2 ± 0.0 | 3.9 ± 0.1**** | 3.3 ± 0.4 | 4.5 ± 1.4 |
|  |  |  |  |  |
