# Supplementary material for: Novel daidzein analogs enhance osteogenic activity of bone marrow-derived mesenchymal stem cells and adipose-derived stromal/stem cells through estrogen receptor dependent and independent mechanisms
Source: Stem Cell Res Ther. 2014 Aug 28;5(4):105. doi: 10.1186/scrt493 (PMC4355363; doi:10.1186/scrt493)
Supplement: Supplementary file 10 — Additional file 10: Presents the gene expression profile of BMSCs and ASCs treated with analog 2l in the presence and absence of fulvestrant on day 14. Data normalized to vehicle-treated cells after 14 days. *P < 0.05; relative to the respective vehicle-treated cells. (DOC 31 KB) [file 13287_2014_413_MOESM10_ESM.doc]

| Gene Name | BMSCs | | ASCs | |
| --- | --- | --- | --- | --- |
|  | - Fulvestrant | + Fulvestrant | - Fulvestrant | + Fulvestrant |
|  |  |  |  |  |
| DMP1 | 3.9 ± 2.0* | 2.4 ± 0.9* | 1.1 ± 0.1 | 1.5 ± 0.3 |
| SOST | 2.2 ± 0.7* | 0.0 ± 0.0 | 1.1 ± 0.3 | 1.1 ± 0.5 |
|  |  |  |  |  |
